# Dual-color dynamic anti-counterfeiting labels with persistent emission after visible excitation allowing smartphone authentication

**DOI:** 10.1038/s41598-022-05885-6

**Published:** 2022-02-08

**Authors:** Ngei Katumo, Kai Li, Bryce S. Richards, Ian A. Howard

**Affiliations:** 1 Institute of Microstructure Technology, Karlsruhe Institute of Technology, Hermann-von-Helmholtz-Platz 1, 76344 Eggenstein-Leopoldshafen, Germany; 2 Light Technology Institute, Karlsruhe Institute of Technology, Engesserstrasse 13, 76131 Karlsruhe, Germany

**Keywords:** Optical materials and structures, Materials for optics

## Abstract

A significant impediment to the deployment of anti-counterfeiting technologies is the reliance on specialized hardware. Here, anti-counterfeiting labels are developed that are both excited and detected using a smartphone. The persistent luminescence pattern and color changes on the timescale of hundreds of milliseconds to seconds. The labels can be authenticated by comparing still images from the red and green channels of video acquired at known times after flashlight excitation against expected reference patterns. The labels are based on a green-emitting SrAl_2_O_4_: Eu^2+^,Dy^3+^ (SAED), and red-emitting CaS:Eu^2+^ phosphors whose lifetimes are varied: (i) for SAED from 0.5 to 11.7 s by annealing the commercial material in air; and (ii) CaS:Eu^2+^ from 0.1 to 0.6 s by varying the dopant concentration. Examples of anti-counterfeiting labels exhibiting changing emission patterns and colors on a seven-segment display, barcode, and emoji are demonstrated. These results demonstrate that phosphors with visible absorption and tunable persistent luminescence lifetimes on the order of hundreds of milliseconds to seconds are attractive for anti-counterfeiting applications as they allow authentication to be performed using only a smartphone. Further development should allow richer color shifts and enhancement of security by embedding further covert anti-counterfeiting features.

## Introduction

Counterfeiting is increasing globally, with the projected financial losses to the global economy projected to hit USD 4.7 trillion in 2022 and at the cost of 5 million legitimate jobs^1^. In addition to the economic consequences, counterfeiting poses major risks to human health and national security. Therefore, there is significant interest in developing anti-counterfeiting technologies. Currently, research into anti-counterfeiting technologies can be categorized into two major classes. Firstly, there is a significant effort on the development of unique unclonable labels, also known as physical unclonable functions^2–5^. These labels should provide the highest level of security. Each label is unique due to a stochastic process in manufacturing, and in practice unclonable, as other manufacturing methods are similarly limited by such stochastic processes and cannot accurately reproduce a given label. Thus, a unique and unclonable label can be attached (in an unremovable fashion) to each product to make duplication impossible. However, there are significant overheads associated with the implementation of such a solution, as each unique label must be characterized and stored in a database to allow authentication^5^. The characterization and storage of information for a large number of unique labels takes time and comes at a cost. Naturally, the losses incurred by manufacturers will need to exceed a certain “pain” threshold before unique unclonable labels will be considered to be adopted as the preferred solution.

The second class of anti-counterfeiting technologies are based on interchangeable labels that are less secure, but are easily implementable, thus representing a lower threshold option. In this direction, interchangeable anti-counterfeiting labels are created with designed overt and covert features that are the same for each label of a given design. In comparison to the unique labels, the costs and time associated with characterizing every label are removed, as each label has the same anti-counterfeiting features that can be communicated to the end-users for authentication (note that the end-users are trusted, as they wish to honestly assess the authenticity of the product in question). However, given enough effort and time to access the unique materials or manufacturing processes, these labels could be forged by counterfeiters. Examples of the features found in the interchangeable anti-counterfeiting labels are watermarks, printed structures, security threads, holograms, two-dimensional codes, and luminescent markers already in common usage for security such as in banknotes, identity cards, and product brands^6,7^. In this paper, the focus is on interchangeable anti-counterfeiting labels, which utilize covert and overt features offered by persistent luminescence markers that are excitable and detectable using a single smartphone—with no other hardware being necessary (e.g. optical filters or lenses).

Luminescence-based anti-counterfeiting tags can be further classified into single-level, double-level, and multilevel according to the level of security provided by the tags^7^. Examples of these are summarized here, but the interested reader is also directed to a recent review by Yu and coworkers^7^. Single-level luminescence anti-counterfeiting tags display the anti-counterfeiting features directly after being stimulated by the appropriate stimuli such as ultraviolet (UV) light or chemical stimuli. These features are usually observable patterns in the anti-counterfeiting label. This level is widely used in certificates and currencies, although relatively susceptible to counterfeit reproduction^7^. The single-level luminescence anti-counterfeiting can use down-shifting (DS) photoluminescence^8,9^ up-conversion (UC) photoluminescence^10–12^, chemiluminescence^13^, or mechanoluminescence^14–16^ features of phosphor materials. Dual-level luminescence-based anti-counterfeiting tags extend upon the single-level tags in that the observable covert and overt features are parameterized and quantified in terms of both the stimuli and the observed stimuli responses. The double level modes are realized through either regulating the excitation source^17–19^ or co-regulating the excitation source with other parameters such as the luminescence lifetime^18^, along with thermal^17^, mechanical and chemical response of the labels^7^. The examples involving regulation of the excitation sources include dual-mode excitation leading to controllable multiple photoluminescence (PL) emission via either UC, DS, or a combination of both. The subsequent co-regulation involves exploiting the other optical responses of the phosphor with respect to diversified excitation and stimulation sources. A broader discussion on the exploitation of the luminescence lifetime feature is provided in the following section as it is the focus of this paper. Finally, multi-level anti-counterfeiting labels are labels whose covert feature authentication affords more than two dimensions of security^7^. For example Sun et al*.* demonstrated multi-mode anti-counterfeiting nano-taggants based on Yb^3+^-, Nd^3+^-, and Ce^3+^-sensitized NaY(Gd)F_4_ nanoparticles, which displayed different colors when the excitation wavelength was changed^19^. The observed emitted color (red, blue, and/or green) was dependent on the excitation wavelength which was either 980 nm, 808 nm, and 254 nm^19^. Other approaches to realize multi-level anti-counterfeiting include power-dependent excitation leading to diversified emission signals^20^, and PL lifetimes from the multiple excitation sources of the labels^21^.

The discussion is now focused on double-level anti-counterfeiting labels that, beyond the simple presence of luminescence, utilize the PL lifetime—in this case the persistent luminescence lifetime—to add a further feature to the labels and improve their anti-counterfeiting security. For example, early work on utilizing PL lifetime in an anti-counterfeiting capacity included forensic work comparing legitimate and counterfeit U.S. dollar bills in terms of the sub-nanosecond PL lifetime of the paper after UV excitation using time-correlated single-photon counting (TCSPC)^22^. Although it was demonstrated that legitimate bills had different lifetimes to fake bills, the cost of the required TCSPC apparatus makes it impractical for widespread application. The lifetime of specific fluorescence materials can also be used for anti-counterfeiting. For example, Kalytchuk et al*.* utilized two different sets of carbon dots with fluorescence lifetimes of 4.4 ns and 6.1 ns respectively after 365 nm UV light excitation to demonstrate anti-counterfeiting tags wherein hidden patterns are revealed by fluorescence lifetime imaging (FLI)^23^. These are very interesting anti-counterfeiting inks, but to image the lifetime on these short time scales expensive hardware and TCSPC is necessary. Lu et al*.* employed luminescent nanocrystals to develop phosphors with emission lifetimes in the 26–662 µs range usable for multi-level anti-counterfeiting and optical coding^24^. Although this presented a significant step to longer lifetimes use in anti-counterfeiting, either a scanning single pixel fast detector or an expensive high-speed camera is still required to detect patterns.

The bulky and expensive equipment needed to image of such nano- to micro-second lifetimes can be avoided by shifting the lifetimes of the labels to the 100 ms to 10 s range^25^. The upper limit on lifetime here is imposed to allow sufficiently rapid authentication, limiting the charging time and video recording time such that authentication can be completed within around 15–20 s. Such extension in the PL lifetime would allow the use of a video captured with a standard camera, such as those in smartphones, to characterize the lifetimes and provide a series of images at different lifetimes^25–27^. Several phosphors exhibiting persistent color change have been explored, but, to date, tailoring lifetimes to create a materials palette optimized for smartphone excitation and detection has not been demonstrated. The work by Liu et al*.* demonstrated the use of ultra-long lifetimes (up to 350 ms) using carbon dots in zeolite matrices with a photoluminescence quantum yield (PLQY) of up to 52% allowing post UV excitation observation with the naked eye^18^. The authors’ previous work advanced on this approach by developing smartphone-camera-readable dynamic anti-counterfeiting labels based on Gd_2_O_2_S: Eu^3+^/Ti^4+^ phosphor^26^. Dynamic patterns were realized via the Ti^4+^ co-doping that allowed the persistent lifetime to be tuned from 1.2 to 5.6 s^26^. However, for these labels, a UV light source was required for exciting the labels before video-recording the emission for subsequent PL-lifetime analysis. In this paper, the use of the smartphone is extended to allow both the excitation and detection of dynamic and color-tunable anti-counterfeiting labels.

Anti-counterfeiting systems that can rely solely on the use of smartphones as that hardware component in an authentication strategy are attractive and convenient as smartphones are ubiquitous^5,25–29^. However, such systems would need to meet several requirements to permit the reliance on only a smartphone being used and no additional hardware being required for the complete authentication process. The first requirement is that the phosphor material used in the anti-counterfeiting label should sufficiently absorb in the visible region (the smartphone flashlight has a 450 nm peak and a broad visible region extending up to 750 nm). Secondly, there should be sufficient emission from the excited anti-counterfeiting material that can be detected with the smartphone camera in a darkened environment. Thirdly, to achieve a dynamic shift in the emission pattern of the anti-counterfeiting labels should contain phosphors with varied persistent lifetimes as already demonstrated in the authors’ previous work^26^. Finally, to achieve a shift in color in the dynamic pattern, a blend of two or more phosphors with different emission spectra is required.

**Figure 1:**
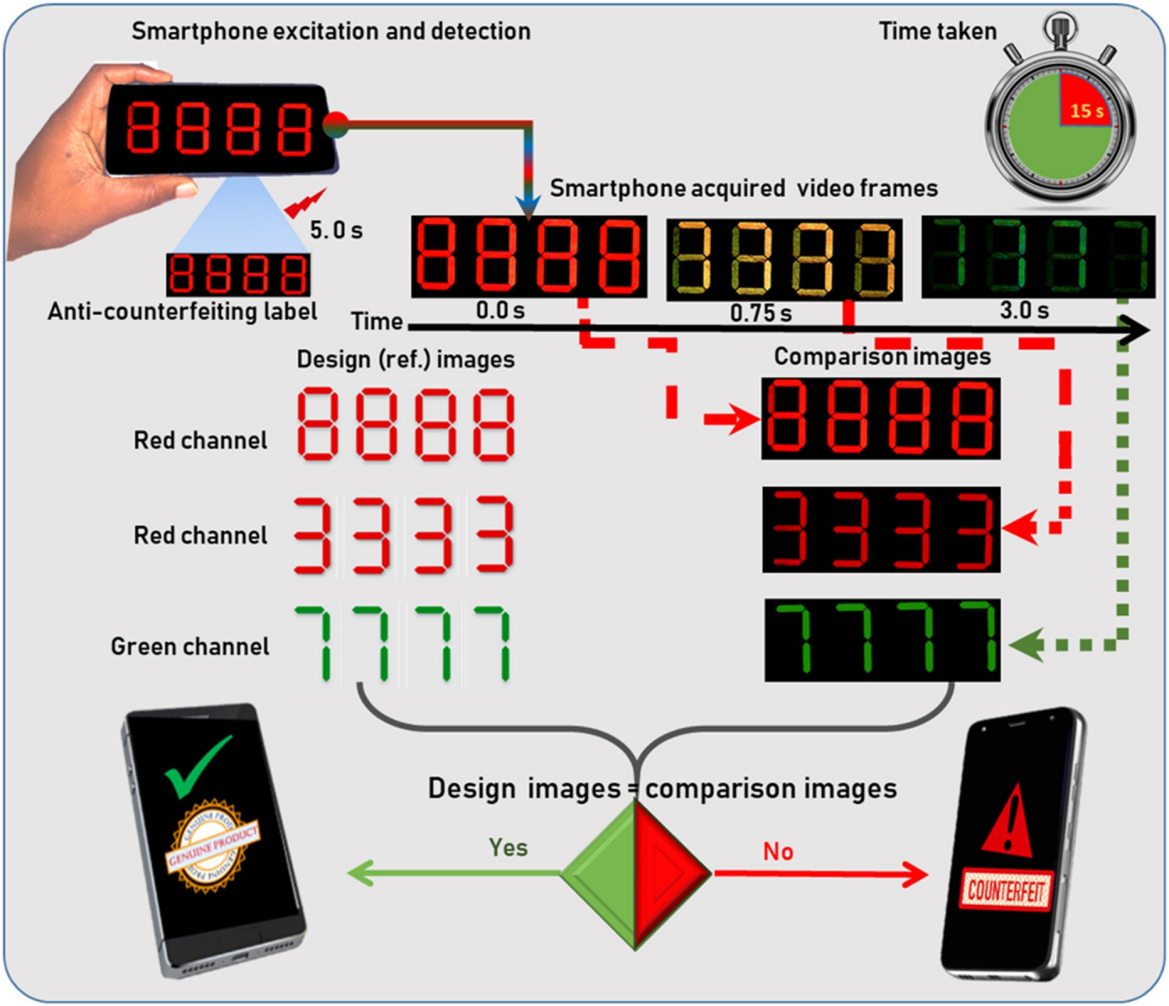
Schematic illustration of smartphone-based authentication of dynamic and color tunable anti-counterfeiting labels. The label is first irradiated with the smartphone-flashlight for 5 s and then the persistent emission is video-recorded with the smartphone’s camera. The acquired raw video is then split at into the red and green channel and the frames at various timestamps (for red 0.0 and 0.75 s and for green 3.0 s) are compared to the intended design images. If the images of the split frames match with the designed image the product is genuine, else counterfeit. The total time taken is less than 15 s.

The smartphone-only authentication process would then proceed as illustrated in Fig. 1. The process starts with the smartphone video-recording turned on, followed by a 5 s flashlight illumination and continuous capture of the emission for a further 10 s. In this case, a seven-segment display is targeted that should have the following characteristics. During illumination with the smartphone flashlight, a ‘8 8 8 8’ should be observed when the red channel of a still frame of the smartphone video is considered. At 0.3 s after the flashlight is extinguished a ‘3 3 3 3’ should be observed in the red channel of the still frame extracted from the video. Finally, 3 s after excitation ‘7 7 7 7’ should be observed in the green channel. If analysis of stills extracted from the smartphone video confirms these expectations, then the label can be deemed authentic.

Herein, dual-color dynamic anti-counterfeiting patterns are realized using blends of green-emitting strontium aluminate doped with europium and dysprosium (${{\text{S}}{\text{r}}_{0.95}{\text{A}}{\text{l}}_{2}{\text{O}}}_{4}\text{:}{\text{Eu}}_{0.02}^{2+}\text{, }{\text{Dy}}_{0.003}^{3+}$ abbreviated as SAED) and red-emitting europium doped calcium sulfide (CaS:Eu^2+^) and strontium sulfide (SrS:Eu^2+^). For the green-emitting SAED phosphor, the Eu^2+^ ion acts as the emitting center. Oxygen vacancies and Dy^3+^ dopants act as electron-trapping centers after photoexcitation and the gradual thermal release of electrons from these traps is responsible for the persistent luminescence^30,31^. The SAED phosphor was annealed in air at various temperatures to reduce its persistent lifetime, tailoring it appropriately for the present anti-counterfeiting application^32^. The SAED phosphors are labeled according to the annealing temperature used (units of °C), for example, phosphors annealed at 700 °C,800 °C, 810 °C and 820 °C are named SAED (700), SAED (800), SAED(810) and SAED (820), respectively. The reference commercial phosphor was named SAED (Ref.). For the red phosphors, the persistent luminescence lifetime of the synthesized ${\text{Ca}}_{1-{\rm x}}\text{S:}{\text{Eu}}_{\text{x}}^{2+}$ and ${\text{Sr}}_{1-{\rm x}}\text{S:}{\text{Eu}}_{\text{x}}^{2+}$ could be varied from 120 to 600 ms by controlling the Eu^2+^ doping from 0.0005 to 0.015 mol. These phosphors are also extensively studied owing to their good optical characteristics viable for applications in bio-imaging^33^, displays^34^, persistent emission^35^ and white-light-emitting diodes^36,37^. Exciting the broad charge-transfer absorption band with blue light leads to a deep-red emission peak centered at 650 nm and 610 nm for CaS:Eu^2+^ and SrS:Eu^2+^, respectively^35,38^. Importantly, Shi et al*.* have studied the persistent luminescence of SAED, and CaSrS_3_:Eu^2+^ (alongside CaAl_2_O_4_:Eu^2+^) and demonstrated excellent properties of these trichromic phosphors for anti-counterfeiting, including color shifts in persistent emission on the timescale of tens of seconds after UV excitation^39^. This is used as a launching point to demonstrate and quantify how persistent lifetimes and color changes can be controlled on the sub-second scale after excitation with a smartphone flashlight in a series of materials based on these hosts, and how the video shot by the same smartphone's camera can be used to authenticate anti-counterfeiting labels based on the developed material palette.

## Results and discussion

**Figure 2:**
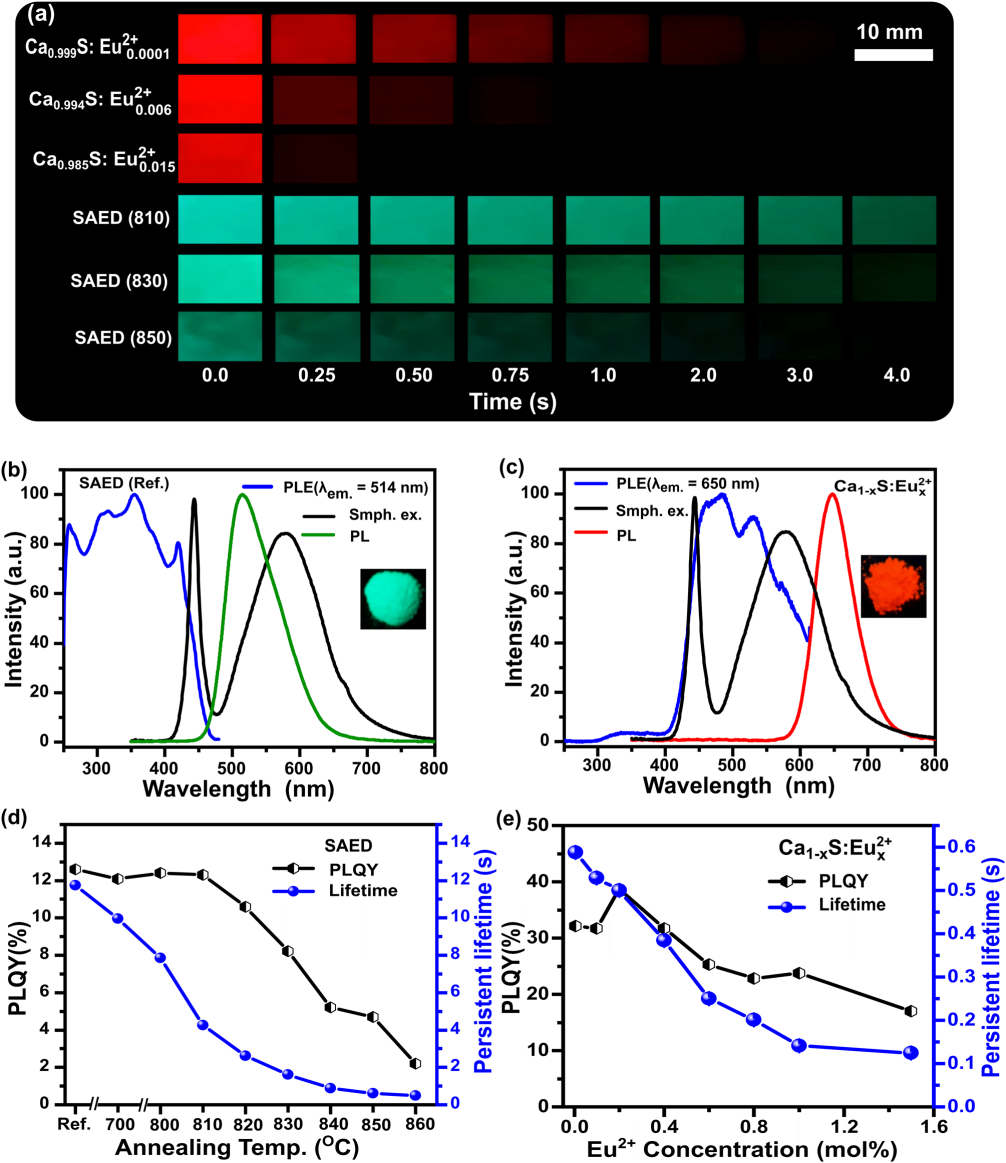
Photoluminescence (PL) properties of the persistent phosphors. **(a)** Video still frames at indicated times after smartphone video excitation is stopped for red phosphors (${\text{Ca}}_{0.999}\text{S:}{\text{Eu}}_{0.001}^{2+}$, ${\text{Ca}}_{0.994}\text{S:}{\text{Eu}}_{0.006}^{2+}$ and ${\text{Ca}}_{0.985}\text{S:}{\text{Eu}}_{0.015}^{2+}$) and green phosphors (SAED (810), SAED (830) and SAED (850)) visually demonstrating the change in persistent lifetime. **(b)** The PL excitation spectrum (monitored at 514 nm) of SAED (Ref.) alongside the emission spectrum of a smartphone-flashlight (Smph.ex.) (Black line) and the PL emission spectra after smartphone excitation. The small overlap of the smartphone excitation with the absorption is just sufficient to allow smartphone excitation. **(c)** The PL excitation spectrum (monitored at 640 nm) of CaS:Eu^2+^ alongside its PL emission spectrum and that of a smartphone-flashlight. The substantial overlap in the phosphor absorption and smartphone-flashlight means that it is efficiently excited. The insets in **(b,c)** show the phosphor emissions after the smartphone excitation. **(d)** The steady state PL quantum yield (PLQY) following 450 nm excitation and persistent lifetime values of SAED samples that are annealed in air for 1 h. **(e)** The steady state PLQY and persistent lifetime values of ${\text{Ca}}_{1-{\rm x}}\text{S:}{\text{Eu}}_{\text{x}}^{2+}$ samples for 0.0005 ≤ x ≤ 0.015 mol after 450 nm LED excitation.

To achieve labels such as those shown in Fig. 1, a key requirement is to use persistent phosphors that are excitable by the smartphone flashlight and have the emission in the visible region. The optical properties of the dynamic anti-counterfeiting phosphor materials demonstrating satisfaction of smartphone flashlight excitation, emission detection, and the material’s varied PL lifetimes are illustrated in Fig. 2. In Fig. 2a smartphone-video-recorded persistent decay frames are displayed of ${\text{Ca}}_{0.999}\text{S:}{\text{Eu}}_{0.001}^{2+}$, ${\text{Ca}}_{0.994}\text{S:}{\text{Eu}}_{0.006}^{2+}$ ${\text{Ca}}_{0.985}\text{S:}{\text{Eu}}_{0.015}^{2+}$ SAED (810), SAED (830), and SAED (850) following smartphone flashlight excitation. For the red-emitting phosphor, the initial emission at time zero is uniformly bright (due to the high PLQY as later demonstrated), but the subsequent frames demonstrate the varied persistent lifetimes that decrease with the increase in Eu^2+^ doping. It is clear from the frames that the ${\text{Ca}}_{0.999}\text{S: }{\text{Eu}}_{0.001}^{2+}$ exhibits a longer red persistent luminescence decay that is observable up to 2 s, whereas the ${\text{Ca}}_{0.985}\text{S: }{\text{Eu}}_{0.015}^{2+}$ exhibits a very short persistent luminescence decay observable up to only 0.25 s post excitation. The effect of annealing the SAED phosphors in the air is also clearly demonstrated following the smartphone flashlight excitation. Notice that the initial intensity of SAED (810) and SAED (830) at time zero are higher compared to SAED (850) and that the persistent luminescence decrease with an increase in the annealing temperature.

Fundamentally, to excite the phosphors with the smartphone flashlight, the absorption (PL excitation) spectra of the phosphors must coincide with the flashlight spectra. Most smartphone flashlights are based on an indium gallium nitride light-emitting diode (LED), with an emission peak at 450 nm^40^. Some of the blue light is converted to longer wavelengths by phosphors in the coating of the LED which leads to the observed white light color. An exemplary spectrum of a smartphone-flashlight obtained using a spectrometer (Thorlabs CCS200) coupled with an optical fiber of 1 mm diameter (Thorlabs FP100URT) is shown in Fig. 2b,c. The excitation spectra coincide with the absorption tail of the SAED phosphors (monitored at 514 nm) that extends from 250 to 450 nm due to 4f^7^–4f^6^5d^1^ transition of Eu^2+^ ion^41^, as displayed in Fig. 2b. Exciting the absorption band with the smartphone flashlight leads to the observed green broadband emission centered at 514 nm due to 4f^7^–4f^6^5d^1^ transition of Eu^2+^. Unlike the SAED phosphor, where smartphone-flashlight excitation is absorbed only by the tail of the absorption spectra, the CaS:Eu^2+^ phosphor PL excitation spectra (monitored at peak emission of 650 nm) extends up to 600 nm, effectively coinciding with the smartphone-flashlight spectra as plotted in Fig. 2c. Smartphone excitation of this absorption band leads to the observed red broadband emission centered at 650 nm. Similar data for the ${\text{Sr}}_{1-{\rm x}}\text{S: } {\text{Eu}}_{\text{x}}^{2+}$ phosphor, also showing a visible broadband absorption extending up to 575 nm (monitored at 614 nm) for which smartphone-flashlight excitation leads to a broadband Eu^2+^ emission centered at 614 nm is shown in Supplementary Fig. S1.

Note that while the three phosphors (SAED, ${\text{Sr}}_{1-{\rm x}}\text{S:}{\text{Eu}}_{\text{x}}^{2+}$ and ${\text{Ca}}_{1-{\rm x}}\text{S:}{\text{Eu}}_{\text{x}}^{2+}$) have Eu^2+^ ion as the emitter, different PL excitation and emission spectra are realized since the 4f^7^ (^8^S_7/2_) → 4f^6^5d^1^ transition of the Eu^2+^ emitter is strongly affected by the crystal field strength and host material^42,43^. The x-ray patterns showing that the phosphors were polycrystalline are also provided in Supplementary Figs. S2–S4 of the supporting information. The refiring in air did not lead to significant structural changes in the SAED. The increasing concentration of Eu^2+^ in CaS does lead to a slight expansion of the lattice noticeable in the XRD data (Fig. S3) as expected for the significant increase in ionic radius of six coordinated Eu^2+^ (1.17 Å) versus Ca^2+^ (1.00 Å). This is not so for the Sr^2+^ case whose 1.18 Å ionic radius that matches much better with the Eu^2+^ substitute. Further discussion is with the data in the supplementary information. The scanning electron microscope images showing large-grained, highly agglomerated, and non-uniform microstructures of the SAED, ${\text{Sr}}_{1-{\rm x}}\text{S:}{\text{Eu}}_{\text{x}}^{2+}$ and ${\text{Ca}}_{1-{\rm x}}\text{S:}{\text{Eu}}_{\text{x}}^{2+}$) are given in Supplementary Fig. S5.

The steady state PLQY and persistent luminescence of the SAED phosphors and the SAED phosphors were greatly affected by the annealing step as shown in Fig. 2d. The PLQY of the phosphors was determined according to the de Mello method^44^, following the phosphor excitation with a 450 nm LED in an integrating sphere that was fiber-coupled with a spectrometer. The persistent lifetime of the various phosphors was determined according to the procedure outlined in Supplementary Fig. S6. In brief, the persistent lifetime was determined by fitting a region of interest, *t*_*1*_*–t*_*2*_, in the persistent decay curves of the phosphors. The initial fitting point *t*_*1*_ was globally set at 0.2 s while *t*_2_ is the time at which the lifetime extracted from the persistent PL emission video decays to 90%. The phosphors annealed below 830 °C were fitted with a double exponential fit while those annealed above 830 °C together with the red-emitting ${\text{Ca}}_{1-{\rm x}}\text{S:}{\text{Eu}}_{\text{x}}^{2+}$ and ${\text{Sr}}_{1-{\rm x}}\text{S:}{\text{Eu}}_{\text{x}}^{2+}$ phosphors fitted with a single exponential as given in following equation:

1 $$I={I}_{o}+{\sum }_{i=1}^{2}{A}_{i} \mathrm{exp}(-t/{\tau }_{i})$$

where ${\tau }_{i}$ are the characteristic lifetimes, ${A}_{i}$ are their respective coefficients and ${I}_{o}$ is an offset. The average persistent lifetime $\overline{\tau }$ was estimated using:

2 $$\overline{\tau }={\sum }_{i=1}^{2}{A}_{i}\times {\tau }_{i}^{2}/{\sum }_{i=1}^{2}{A}_{i}\times {\tau }_{i}$$

The SAED (Ref.), SAED (700), SAED (800), SAED (810), and SAED (820) green-emitting phosphors registered PLQYs above 11 ± 0.5% while the persistent lifetime decreased from 11.7 s to 2.6 s for SAED (Ref.) and SAED (830) respectively. The annealing step in the air gradually decreased the persistent lifetime, with the SAED (860) phosphor recording a persistent lifetime of 0.5 s and a low PLQY of 2%. The SAED phosphors annealed at 840 °C or greater exhibited a PLQY below 6% and their initial emission intensities were weak (see Fig. 2a SAED (850)). For this reason, they were deemed infeasible for the development of dual-color dynamic anti-counterfeiting labels as later demonstrated. Nevertheless, the SAED annealed at up to 830 °C created a series of green-emitting persistent phosphors with a varying lifetime that is sufficient for the demonstration of dynamic and color-tunable anti-counterfeiting labels authenticatable using a smartphone. The mechanism for the gradual decrease in both persistent luminescence and PLQY after reannealing above 820 °C is considered in the following way. After 450 nm excitation, the persistent emission in SAED is proposed to be from a subset of the substituted Sr sites that can be influenced by nearby Dy^3+^^45,46^. Also, in the absence of Dy^3+^, the oxygen vacancies in the SrAl_2_O_4_ create electron traps that are proposed to contribute to the persistent emission^45^. Although the precise understanding of all the various causes of persistent luminescence in SAED is still being refined^46,47^, given the concurrent reduction of the persistent lifetime and intensity of emission we postulate that a reduction of the oxygen vacancies could lead to reduced trap density and depth, and the reduction in intensity might be related to the previously observed oxidation of the radiative Eu^2+^ to an inactive Eu^3+^ ion in the host^32,48^. One other possibility is that Eu^3+^ could help funnel energy towards NIR emission from the Dy^3+^^49^, but this is ruled out by the observation that the NIR intensity decreases after reannealing (Fig. S7). In addition, the annealing in the air led to the narrowing of the SAED absorption bands as illustrated in Supplementary Fig. S8, however, neither a shift in emission spectra nor any new peaks were observed. Further work is warranted to realize green-emitting persistent phosphors with both high PLQY and strong absorption in the 450 nm region.

The persistent luminescence lifetime and PLQY behavior of the red-emitting phosphor ${\text{Ca}}_{1-{\rm x}}\text{S: } {\text{Eu}}_{\text{x}}^{2+}$ for (x = 0.0005–0.015 mol) is presented in Fig. 2e. The PLQY first increased gradually with Eu^2+^ doping from 32 ± 1% for ${\text{Ca}}_{0.9995}\text{S:}{\text{Eu}}_{0.0005}^{2+}$ up to 39 ± 1% for ${\text{Ca}}_{0.998}\text{S:}{\text{Eu}}_{0.002}^{2+}$. Further doping led to a decline of the PLQY with the 0.015Eu^2+^ doped CaS phosphor registering a PLQY of 18 ± 1%. While the PLQY exhibited a maximum at a concentration of 0.002 mol of Eu^2+^ ions, the persistent lifetime monotonically decreased with an increase in Eu^2+^ doping from 590 ms for ${\text{Ca}}_{0.9995}\text{S:}{\text{Eu}}_{0.0005}^{2+}$ to 125 ms for ${\text{Ca}}_{0.985}\text{S:}{\text{Eu}}_{0.015}^{2+}$ (see Fig. 2e). Again, the persistent lifetime was established via a single exponential fit to Eq. (1), in the fitting region defined by *t*_*1*_—*t*_*2*_ shown in Supplementary Fig. S6. Similarly, in SrS: Eu^2+^, the PLQY remains relatively constant at 37 ± 2% for doping concentrations below 0.008 mol of Eu^2+^ doping but slightly decreases with further doping to 28 ± 1% for 0.015 mol Eu^2+^ doping. In the same doping regime, the delayed persistent luminescence lifetimes reduce from 377 to 150 ms for ${\text{Sr}}_{0.999}\text{S:}{\text{Eu}}_{0.001}^{2+}$ and ${\text{Sr}}_{0.985}\text{S:}{\text{Eu}}_{0.015}^{2+}$ respectively as shown in Supplementary Fig. S9. The persistent lifetime plots of SAED and CaS:Eu^2+^ phosphors are also presented in Supplementary Figs. S10 and S11 respectively and the persistent lifetime data in Supplementary Table S1.

Although a fully conclusive analysis of the mechanism leading to the change in persistent emission lifetime with Eu^2+^ concentration in CaS is beyond the scope of the current work, the first hypothesis to consider is that either (or both) the radiative or (and) the non-radiative channel for the trapped states that lead to the persistent emission are affected by the Eu^2+^ concentration. The rate of the radiative channel for the persistent emission depends on the activation energy required for the trapped state to be stimulated back to an emissive center. Therefore, if there were a change in this radiative mechanism (i.e., a change in the trap depth) caused by the Eu^2+^ concentration, then this could be measured in the dependence of the persistent emission lifetime on temperature. The activation energy for the persistent emission is estimated by fitting the temperature dependence of the persistent emission lifetime for ${\mathrm{Ca}}_{0.999}\mathrm{S}:{\mathrm{Eu}}_{0.001}^{2+}$, ${\mathrm{Ca}}_{0.996}\mathrm{S}:{\mathrm{Eu}}_{0.004}^{2+}$ and ${\mathrm{Ca}}_{0.992}\mathrm{S}:{\mathrm{Eu}}_{0.008}^{2+}$ between 230 and 450 K to a simple Arrhenius form (Figure S12). The activation energy so estimated does not significantly change with the sample series. The activation energy remains small (between 0.05 and 0.1 eV) and constant over the Eu^2+^ concentrations, suggesting that a changing trap depth based on the Eu^2+^ concentration is not responsible for the observed persistent lifetime tuning. This leaves the hypothesis that the non-radiative deactivation of the trapped states becomes faster with increasing Eu^2+^ concentration. This is consistent with the decrease in PLQY at the higher Eu^2+^ concentrations (Fig. 1e) and the temperature dependence of the integrated PL intensity (Fig. S13). Irrespective of the precise mechanism of the persistent lifetime tuning, it is certainly adequate for the presented application.

**Figure 3:**
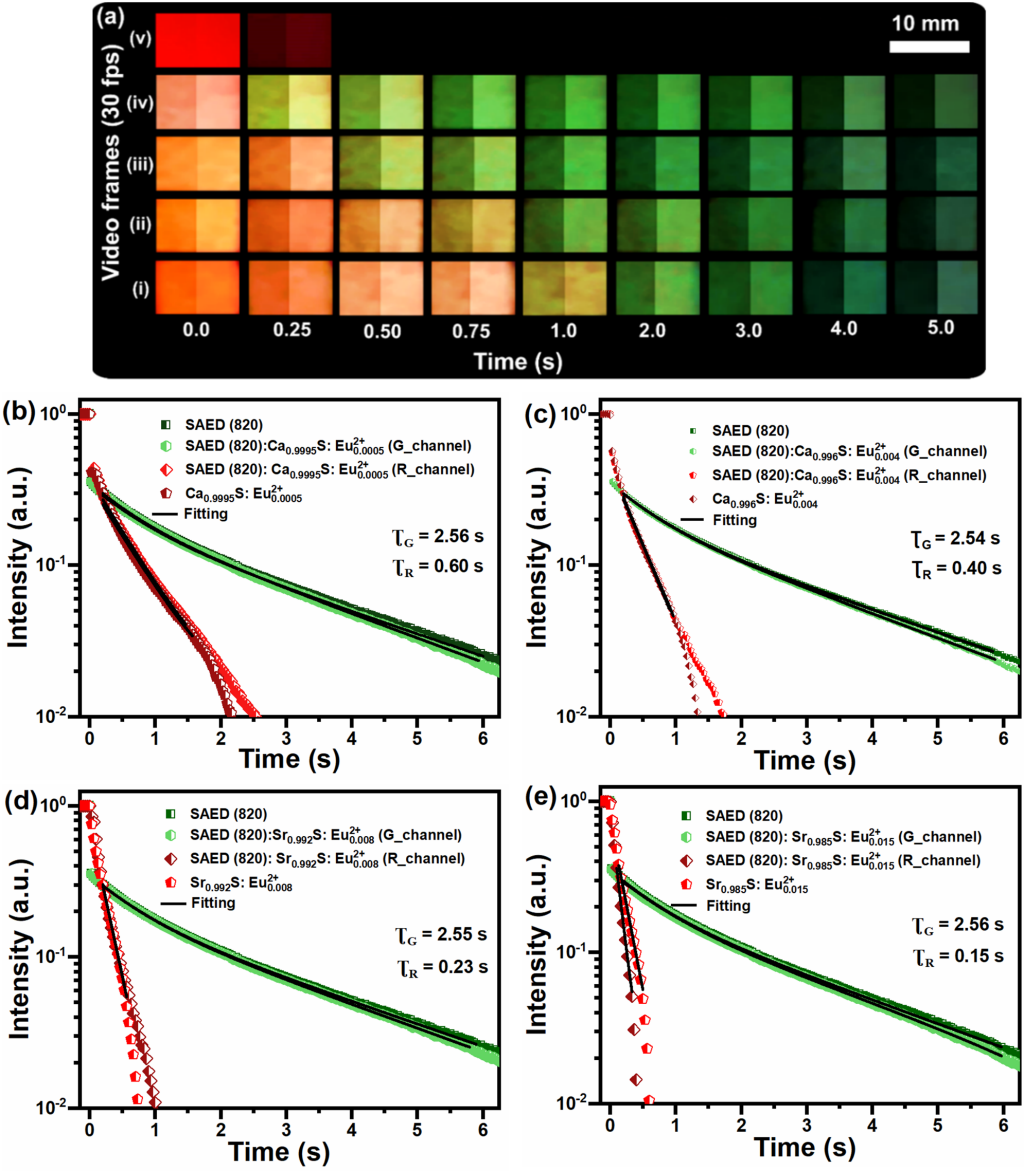
Tuning change of emission color and persistent lifetime of blended phosphors.** (a)** Illustration of color tuning with time using blends of different red and green persistent phosphors. The left half of each image shows the raw video frame (change in color and intensity), the right half highlights the color change only by normalizing to a constant intensity. The compositions are: (i) SAED (820):${\text{Ca}}_{0.9995}\text{S:}{\text{Eu}}_{0.0005}^{2+}$ (ii) SAED (820):${\text{Ca}}_{0.996}\text{S:}{\text{Eu}}_{0.004}^{2+}$ (iii) SAED (820): ${\text{Sr}}_{0.992}\text{S:}{\text{Eu}}_{0.008}^{2+}$ and (iv) SAED (820):${\text{Sr}}_{0.985}\text{S: }{\text{Eu}}_{0.015}^{2+}$ at 95:5 wt.%. The longer the persistent lifetime of the red phosphor the longer the transition takes and vice-versa. The extracted red and green channels persistent lifetime of phosphors: **(b)** SAED (820):${\text{Ca}}_{0.9995}\text{S:}{\text{Eu}}_{0.0005}^{2+}$, **(c)** SAED (820):${\text{Ca}}_{0.996}\text{S:}{\text{Eu}}_{0.004}^{{2}\text{+}}$, **(d)** SAED (820):${\text{Sr}}_{0.992}\text{S:}{\text{Eu}}_{\text{0.008} \, }^{2+}$ and **(e)** SAED (820):${\text{Sr}}_{0.985}\text{S:}{\text{Eu}}_{0.015}^{2+}$ alongside the pure phosphors.

Color-tunability of the persistent emission with time was realized by physically blending the SAED:CaS:Eu^2+^ and SAED:SrS:Eu^2+^ phosphors leading to the realization of red to green emission transition post excitation as shown Fig. 3a. These blended phosphors are composed of SAED (820):${\text{Ca}}_{0.9995}\text{S:}{\text{Eu}}_{0.0005}^{2+}$ for (i), SAED (820):${\text{Ca}}_{0.994}\text{S:}{\text{Eu}}_{0.006}^{2+}$ for (ii), SAED (820):${\text{Sr}}_{0.992}\text{S:}{\text{Eu}}_{0.008}^{2+}$ for (iii) and SAED (820):${\text{Sr}}_{0.985}\text{S:}{\text{Eu}}_{0.015}^{2+}$ for (iv) at a ratio of 95:5 wt% of SAED to the red phosphors. Notice that half of these images are partially saturated for color clarity. The blending ratio of 95:5 wt.% is selected to balance the emission from the CaS and SAED to red provide a gradual transition from red to green (see Fig. S14 for examples of the transition at other ratios). The red to green transition time is dictated by the red phosphor blended with SAED (820). The longer the red persistent lifetime the longer the red to green transition takes and vice-versa. Only the red to green transition could be realized as the initial emission intensity and steady-state PLQY is higher for all the red phosphors compared to the SAED phosphors. For the SAED (820):${\text{Ca}}_{0.9995}\text{S:}{\text{Eu}}_{0.0005}^{2+}$ blend, the emission remains red beyond 0.75 s before the transition to yellowish color at 1 s and finally to green. The shortest red phosphor blend, SAED (820):${\text{Sr}}_{0.985}\text{S:}{\text{Eu}}_{0.015}^{2+}$ on the other hand only shows a prompt red immediately after excitation which then transitions to yellow (green and red) at 0.25 s and finally a dominant green at 0.75 s. A failed color-transition is also demonstrated using SAED (850):${\text{Ca}}_{0.985}\text{S:}{\text{Eu}}_{0.015}^{2+}$ for (v) for which the green emission could not be realized. Note that the SAED (850) had an initial weak emission (see Fig. 2a) as well as being weakly absorbing.

The encoding capacity was expanded by further extracting the color-specific (red and green) persistent lifetimes of the phosphors as illustrated in Fig. 3b–e. The green emission from SAED (820) phosphor registered a persistent lifetime of 2.55 ± 0.01 s across all the four samples, a value representing a 2.3% decline in the average persistent lifetime registered by pure SAED (820) due to re-absorption. On the other hand, the persistent lifetime of the red-emitting phosphors registered persistent lifetimes of 0.60 s, 0.40 s, 0.27 s, and 0.16 s representing 2%, 4%, 4.5% and 7% persistent lifetime increase for ${\text{Ca}}_{0.9995}\text{S:}{\text{Eu}}_{0.0005}^{2+}$, ${\text{Ca}}_{0.996}\text{S:}{\text{Eu}}_{0.004}^{2+}$, ${\text{Sr}}_{0.992}\text{S:}{\text{Eu}}_{0.008}^{2+}$, ${\text{Sr}}_{0.985}\text{S:}{\text{Eu}}_{0.015}^{2+}$ respectively. The red-emitting phosphors with the long persistent lifetime had the least enhancement while the shortest had the largest. The persistent lifetime of the blends is presented in Supplementary Table S2.

The increase in the registered persistent lifetime of the red-emitting phosphors is small enough that it does not pose a problem to designing the color change of labels with the lifetimes expected from the pure material. One explanation of the slightly enhanced persistent lifetime is some limited reabsorption of SAED emission by the CaS:Eu^2+^ and SrS:Eu^2+^ as their absorption band coincides with the SAED emission. The amount of this reabsorption will depend on the precise distribution of phosphors in the labels, with slight spatial separation of the phosphors instead of the intimate mixing presented herein being advantageous to minimize reabsorption. Nonetheless, the data presented in Fig. 3 (and subsequently) demonstrates that even in this case reabsorption is minor and that the persistent lifetimes of the red and green emission in blended materials remain close to the lifetimes of the pure red and green materials.

**Figure 4:**
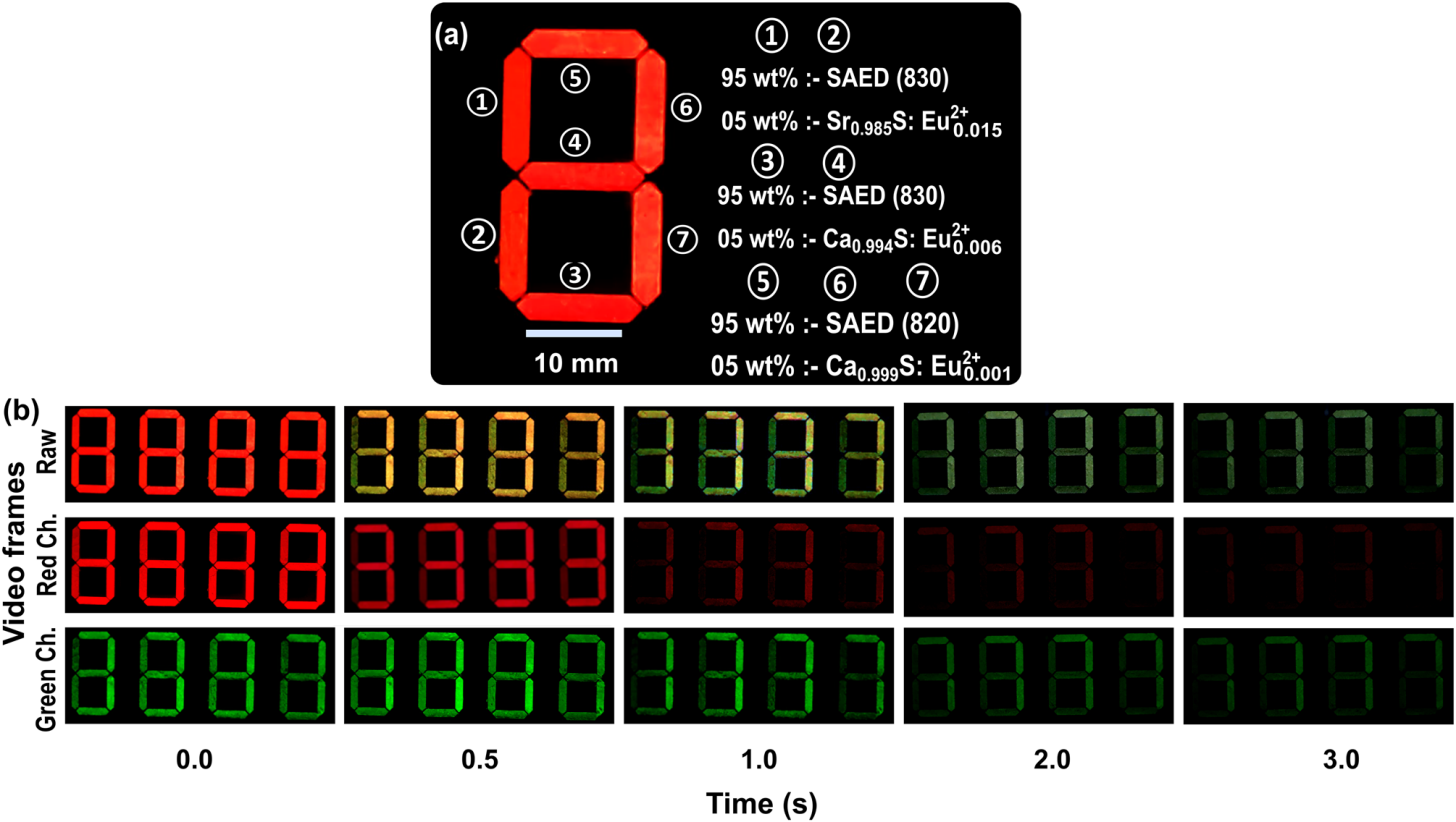
Demonstration of color and pattern changing persistent luminescence anti-counterfeiting labels following smartphone excitation. **(a)** Schematic representation detailing the phosphors placed in each of the 7-segment display. **(b)** Smartphone acquired video frames of interest for four digits of the 7-segment display described in **(a)** after 5 s excitation with smartphone-flashlight. The raw video frames are then split in to the red and green channels. The raw video transitions from ‘8 8 8 8’ in red, to a yellowish (red and green) ‘3 3 3 3’ and finally to a green ‘7 7 7 7’ at 0.5 s and beyond 1 s respectively. Exploring the red channels reveals that the initial ‘8 8 8 8’ in red, transitions to a ‘3 3 3 3’ and finally to a red ‘7 7 7 7’ at 0.5 s and beyond 1 s respectively. The first and second transition is due to exhaustion of majority of the red emission emanating from ${\text{Sr}}_{0.985}\text{S:}{\text{Eu}}_{0.015}^{2+}$ and ${\text{Ca}}_{0.994}\text{S:}{\text{Eu}}_{0.006}^{2+}$ in segments ①—② and ③—④ respectively. This is different from the green channel transition where the initial red-channel-concealed ‘8 8 8 8’ transitions directly to a green ‘7 7 7 7’ at 0.5 s as the segments ①—④ contained on SAED (830) and ⑤—⑦ has SAED (820).

To demonstrate dynamic and color-tunable anti-counterfeiting phosphors, labels were developed by embedding the blended phosphors in a four-digit seven-segment display as shown in Fig. 4a. Each of the 7-segments of a digit contained 0.040 ± 0.005 g. The dynamic and color-tunable label following smartphone excitation is illustrated by the extracted video frames (raw) at the various timestamps together with the coinciding red and green channel frames obtained by splitting the original raw frames as shown in Fig. 4b. Immediately after the smartphone flashlight is turned off, the four digits display ‘8 8 8 8’ in red. From the raw video, it’s imperative to say that the pattern then changes to a yellowish ‘3 3 3 3’ display at 0.5 s after which it changes again to a green ‘7 7 7 7’ after 1 s respectively. The yellowish ‘3 3 3 3’ display occurs when segments ① and ② exhaust most of the red emission while the final ‘7 7 7 7’ display is due to segments ③ and ④ exhausting most of their red emission.

The anti-counterfeiting label covert features are revealed when the raw video frames are split into red and green channels. For the red channel, the color transitions from the initial red ‘8 8 8 8’ display to a ‘3 3 3 3’ red at 0.5 s after the red emission in segments ① and ② gets exhausted. Afterward, the display for this red channel changes to ‘7 7 7 7’ after the red emission from segments ③ and ④ in each digit gets exhausted. On the other hand, the green channel, not observable at time zero for the raw video, but observable only after channel splitting, changes from a green ‘8 8 8 8’ directly to a ‘7 7 7 7’ after 0.5 s. Note that only two green phosphors are used, with the longer SAED (820) only being used intentionally to lead to the intended ‘7 7 7 7’ display in segments ⑤—⑦. For smartphone anti-counterfeiting applications, the change of the green channel from ‘8 8 8 8’ channel directly to a ‘7 7 7 7’ after 0.5 s concealed by the red emission for instance can act as the covert feature. In addition, multi-level intricacy could be realized from the analysis of the persistent lifetime of the blended phosphors. These observable changes are similar to the persistent decay of the independent phosphors forming the blends. The persistent lifetime of the phosphor blends is presented in Supplementary Fig. S16. The persistent lifetime for the red-emitting phosphors ${\text{Sr}}_{0.985}\text{S:}{\text{Eu}}_{0.015}^{2+}$ and ${\text{Ca}}_{\text{0.}{994}}\text{S:}{\text{Eu}}_{0.006}^{2+}$, used in the blends were determined as 0.16 s and 0.27 s, respectively. The SAED (830) extracted persistent lifetime was determined to be 1.56 s which is a 3.7% decrease from the persistent lifetime of the pure phosphor.

**Figure 5:**
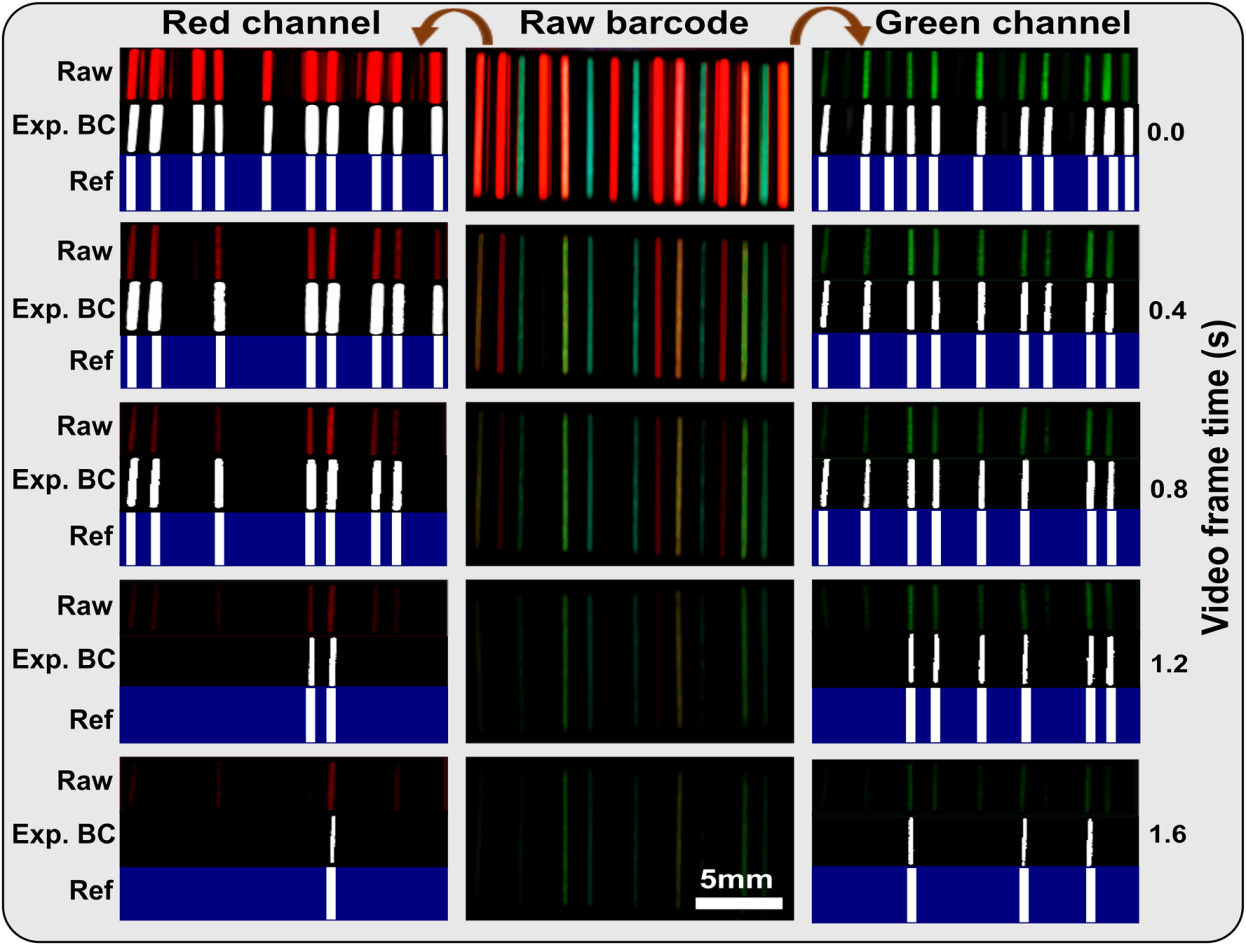
A multi-color-tunable 15-line dynamic barcode registering different dynamic codes following evaluation of the red and green channel of the emission. From left to right the raw barcode lines have the following samples SAED (850) in line 11, SAED (840) in line 3, SAED (830) lines 6, 8 and 14, ${\text{Ca}}_{0.999}\text{S:}{\text{Eu}}_{0.001}^{2+}$ (lines 9), ${\text{Ca}}_{0.996}\text{S:}{\text{Eu}}_{0.004}^{2+}$, (lines 2 and 12), ${\text{Ca}}_{0.9}\text{S:}{\text{Eu}}_{0.01}^{2+}$ (lines 7), and ${\text{Sr}}_{0.99}\text{S:}{\text{Eu}}_{0.01}^{2+}$, in line 4, and ${\text{Sr}}_{0.994}\text{S:}{\text{Eu}}_{0.006}^{2+}$, line 15. Blended phosphors shown in Fig. 3b include phosphors (i) in line 10, phosphor (ii) in line 5 and line 13. The barcodes remain on (‘1’) when above the threshold parameter and off (dark) when below after the splitting of the red and green channels. For each barcode, the raw barcode is shown alongside the processes barcode and the reference barcode for authentication at the various time stamps.

To enhance the concealment level and increase the number of parameters at disposal to optically code the anti-counterfeiting labels, dynamic and color-tunable anti-counterfeiting barcodes were developed based on the single phosphors and blended phosphors as shown in Fig. 5. The barcodes were developed using glass tubes with an internal diameter of 1 mm and were 10 mm long. The 1 mm internal diameter provided sufficient thickness to accommodate the weak absorption of the SAED phosphors. The phosphor utilized in each bar, from left to right is as described in the caption. At a ratio of 95:5 wt.% a single barcode had 0.025 ± 0.005 g. The raw barcode in the middle shows the video frames following smartphone flashlight irradiation which are then split into the red and green channels respectively. If the pixels for a channel fall below 12% (~ 30 pixels out of 255 possible pixels per channel) then the bar transition from one (bright white lines) to zero (transition to black) for that channel. The registered barcodes are re-confirmed from the information already obtained from the persistent luminescence decay of the phosphors that is also provided as a ref alongside the frames. At time zero, the red channel barcode reads ‘110110101111101’ and this transitions to ‘110010001111101’ then to ‘110010001101100’ then ‘000000001100000’ and finally to ‘000000000100000’ at 0.4, 0.8, 1.2, and 1.6 s respectively. On the other hand, the green channel barcode at time zero is ‘101111010110111’ and transitions to ‘101011010110110’ and then 101011010100110’ and then ‘000011010100110’ and finally to ‘000010000100100’ at times 0.4, 0.8, 1.2, and 1.6 s respectively. The green and red barcode readings and transitions are therefore completely different, and hence uniquely concealing the authentication of the label. The 15-bar dynamic and color-tunable barcode, with four verification timestamps for the red and green channels, for instance, provides for over 40! possible combinations.

**Figure 6:**
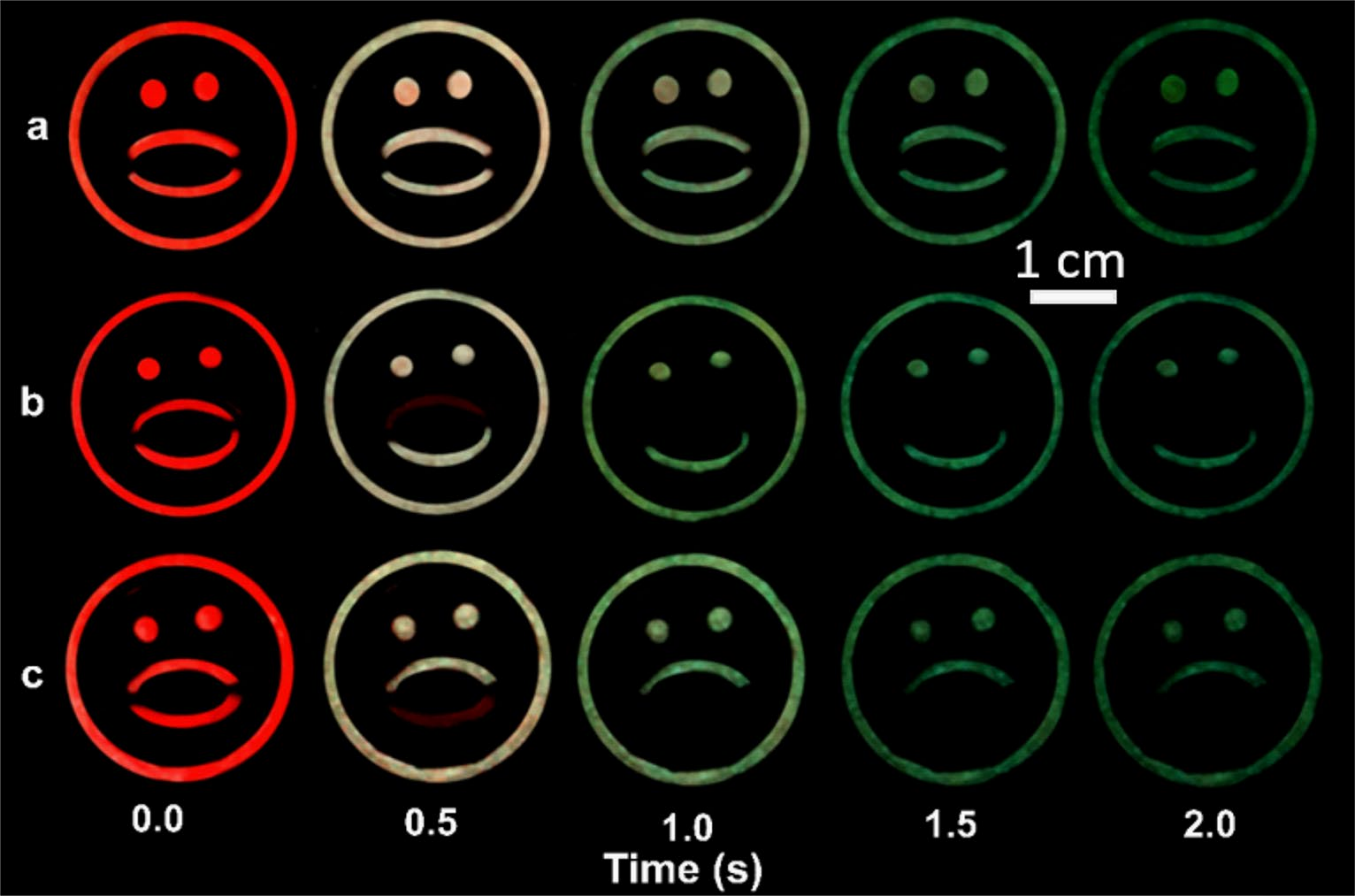
Persistent luminescent emoji images utilizing both color tunability and persistent emission to transform emoji from an initial wow image after smartphone excitation for 10 s. **(a)** The wow emoji color transforms to a green wow emoji. **(b)** The initial red wow emoji transforms to a green smiley face emoji after 1 s. **(c)** The initial red wow emoji transforms to a green sad face emoji after 1 s. The external diameter of the emoji is 3.0 cm.

In a further experiment, the graphical encryption based on dynamic and color-tunable emoji patterns was developed with only two phosphor blends as shown in Fig. 6. The emoji patterns are developed by drop-casting the phosphor blends SAED (820): ${\text{Ca}}_{0.999}\text{S:}{\text{Eu}}_{0.001}^{2+}$ and SAED (900):${\text{Ca}}_{0.992}\text{S:}{\text{Eu}}_{0.008}^{2+}$ to form the emoji. Immediately after smartphone excitation, all the initial images show a ‘wow face’ emoji in red. The ‘emoji face’ is continuously shown in Fig. 6a even when the color changes from red to green after 1 s as this pattern is based on the SAED (820):${\text{Ca}}_{0.999}\text{S:}{\text{Eu}}_{0.001}^{2+}$ phosphor blend only. In Fig. 6b, the upper lip of the emoji is replaced with SAED (900):${\text{Ca}}_{0.992}\text{S:}{\text{Eu}}_{0.008}^{2+}$ leading to a green smiley face emoji when the ${\text{Ca}}_{0.992}\text{S:}{\text{Eu}}_{0.008}^{2+}$ red emission in the upper lip and the red emission from the blend are exhausted. The SAED (900) does not emit green due to the high annealing temperature in the air following smartphone flashlight excitation. A similar approach to the lower lip leads to a sad-faced emoji as shown in Fig. 6c. To ascertain that the similar transition can be observed over a wide range of temperatures, videos were also taken at 278 and 318 K (5 and 45 °C). The data shown in Fig. S17 confirm that a similar transition is observed at all temperatures, meaning that the optically unique behavior can be observed over the range of standard ambient temperatures. These images depict the power of dynamic and color-tunable anti-counterfeiting labels as a cheap smartphone verifiable anti-counterfeit label in which the covert graphical feature is observed post excitation.

## Conclusion

Dynamic and color-tunable anti-counterfeiting tags excitable and readable with a smartphone were fabricated using SAED, ${\text{Ca}}_{1-{\rm x}}\text{S:}{\text{Eu}}_{\text{x}}^{2+}$ and ${\text{Sr}}_{1-{\rm x}}\text{S:}{\text{Eu}}_{\text{x}}^{2+}$ phosphors. The absorption and emission bands of the phosphors were in the visible region, and this allowed a smartphone flashlight to excite them after which the smartphones’ camera was used to video-record their persistent emission. For the SAED-based phosphors, it was possible to tune the persistent decay from 0.5 to 12.6 s via annealing them in the air at between 800 and 860 °C. The persistent luminescence decay of ${\text{Ca}}_{1-{\rm x}}\text{S:}{\text{Eu}}_{\text{x}}^{2+}$ and ${\text{Sr}}_{1-{\rm x}}\text{S:}{\text{Eu}}_{\text{x}}^{2+}$ was tuned in the 120–590 ms range by increasing the Eu^2+^ doping concentration from 0.0005 to 0.015 mol. The combination of these phosphors allows labels with various rates of transition from red to green emission color. Various instances of anti-counterfeiting labels based on these phosphor combinations were demonstrated and it was shown that they could be excited and authenticated with a single smartphone. The uniqueness and ease of authentication of these persistent luminescence-based labels is an attractive prospect for application. Further work to develop green-emitting persistent phosphors with better absorption at 450 nm (and high efficiency) would be very beneficial to this field. In addition, reduction of particle size while maintaining efficiency would be of interest to make such phosphors compatible with a larger number of printing techniques. Development of further emission lines, and potentially also a deliberate distortion of the persistent lifetimes of given components due to reabsorption are possible routes to enhancing the security of labels based on this approach.

## Experimental section

### Materials

The raw materials included the commercial strontium aluminate doped with europium and dysprosium, ${{\text{S}}{\text{r}}_{0.95}{\text{A}}{\text{l}}_{2}{\text{O}}}_{4}\text{:}{\text{Eu}}_{0.02}^{2+}\text{, }{\text{Dy}}_{0.003}^{3+}$, (purity 98%, Sigma Aldrich) referred herein throughout as SAED (Ref.). Other materials used include CaSO_4_.2H_2_O (purity, < 99%, VWR—Alfar Aesar), SrSO_4_ (Purity 99%, VWR—Alfar Aesar), Eu_2_O_3_ (purity, 99.99%, Sigma Aldrich), and activated carbon, (C). The materials were used without further purification.

The commercial SAED phosphor was annealed in various temperatures (700 °C, 800 °C, 810 °C, 820 °C, 830 °C, 840 °C, 850 °C, 860 °C, and 890 °C) to tune the persistent luminescence decay. The ramping rate was maintained at 8 °C per minute for all the samples, with the maximum temperature being maintained for one hour after which the phosphors were let to cool to room temperature. The phosphors were then labeled according to the annealing temperature, SAED (700), SAED (800) SAED (810), SAED (820), SAED (830), SAED (840), SAED (850), SAED (860) and SAED (890). The commercial reference phosphor is referred to throughout this document as SAED (Ref.). Phosphors annealed beyond 870 °C had extremely low emissions and were therefore not useful unless used for blending to ensure composite blends look similar.

The series of ${\text{Ca}}_{1-{\rm x}}\text{S: }{\text{Eu}}_{\text{x}}^{2+}$ (0.0005 < x < 0.015 mol) and ${\text{Sr}}_{1-{\rm x}}\text{S:}{\text{Eu}}_{\text{x}}^{2+}$ (0.0005 < x < 0.015 mol) phosphors were prepared via solid-state reaction in a reducing environment using a tube furnace. First, the molar ratio materials were weighed, then two mol of activated carbon were added. The mixture was then thoroughly grounded for 15 min using a mortar. An appropriate amount of pure ethanol (~ 2 ml) was added to each batch to make a clay-composite mold of the materials. The materials were then let to dry and then transferred to the tube furnace and annealed at 1200 °C for 3 h. in a reductive atmosphere (Ar:H_2_ = 95:5) at a ramping rate of 5 °C/min. The phosphors were let to cool to room temperature and then ground to form powders for subsequent characterization.

### Seven segment display development

The four-digit seven-segment display (made of a PMMA sheet) was developed in-house using an ultra-precision milling machine. The seven-segment display numbers were generated using a SURFCAM traditional software. The numbers were based on a 10 mm by 3 mm segment with a depth of 0.5 mm. The segment edges were maintained at 90° for all the segments. The boundary between segments was 0.5 mm. The selected blended phosphors were carefully embedded in the segments pressed to form the display digits.

### Optical barcode development

The dynamic and color-tunable barcodes were developed by filling 1 mm diameter glass tubes with the phosphors and respective blends and aligning them in a 1 mm spacing. The glass tubes were then sealed and arranged in glue-taped black paper to form the dynamic barcodes. The barcodes were then analyzed by exciting them with a smartphone flashlight for 5 s and video-recording the emissions. The videos were then analyzed in MATLAB to derive the persistence luminescence as a function of time.

### Drop casting the graphics

The suspensions were created by first dissolving 5 g of polyvinylpyrrolidone in a 20 ml mixture of ethanol and water (ethanol:deionized water at 30:70 wt.%) and stirring with a magnetic stirrer for 1 h until the fluid was clear. The 20 ml portion was then divided into two portions of 15 ml and 5 ml. One gram of the blended phosphor, SAED (820):${\text{Ca}}_{0.999}\text{S:}{\text{Eu}}_{0.001}^{2+}$ was put in the 15 ml portion and magnetically stirred for 30 min until the mixture gained homogeneity. For the 5 ml portion, 0.33 g of SAED (900):${\text{Ca}}_{0.992}\text{S:}{\text{Eu}}_{0.008}^{2+}$ was added and magnetically mixed for 30 min to obtain a homogeneous mixture. Note that the SAED (900) used is a non-emitting phosphor, purposely added to have the two portions look-alike the naked eye. The blended phosphors were then drop cast freehand on glass slides with the emoji patterns being under the glass. During the process, the glass slides were maintained at 50 °C during the casting stage to speed the drying process.

### Characterization

#### Optical characterization

The PL excitation spectra were recorded using a spectrometer (Varian Carry 50) in the 600–250 nm range, monitored at 650 nm for ${\text{Ca}}_{1-{\rm x}}{\text{S}}\text{:}{\text{Eu}}_{\text{x}}^{2+}$ 614 nm for ${\text{Sr}}_{1-{\rm x}}\text{S:}{\text{Eu}}_{\text{x}}^{2+}$ and 514 nm for the SAED phosphors. The phosphors were placed in 1 mm quartz cuvettes during the measurement. The PL emission was recorded using a spectrometer (CCS200, Thorlabs) coupled with an optical fiber of 1 mm diameter (FP100URT, Thorlabs) after 450 nm LED excitation. To calculate the PLQY, an optical setup with a spectrometer (AvaSpec-HS2048XL, Avantes) coupled with an optical fiber (FP100URT, Thorlabs) to an integrating sphere (15 cm diameter, Lab sphere) was used. A 450 nm LED, controlled with a benchtop diode controller (ITC4001, Thorlabs) was used as the excitation source and was directed towards the sample in quartz cuvettes inside the integrating sphere where several measurements as described by de Mello et al*.* method were taken^44^. Measurements of the empty sphere, direct and indirect excitation spectra, direct and indirect PL emission spectra, and black background were recorded, from which the PLQY was calculated.

### Persistent luminescence lifetime

The persistent lifetime of the phosphors was recorded using a smartphone (Galaxy A5(2017), Samsung Electronics) via a third-party app Open Camera (v1.48.1 Code: 77) found in the Google Play app store. To have control of the exposure and shutter settings, the application programming interface (API) of the app was changed from the default API to camera 2 API. In this mode, we then set the spatial resolution of the camera to 1920 × 1080 pixels, acquisition speed to normal, white balance to fluorescent, and color effect to none. Further, the focus distance was set to 10 cm, the shutter speed set to 1/33 s, (allowing 30 frames-per-second capture), and the ISO to 1450.

The persistent luminescence of the phosphors was then video recorded as follows. The phosphors were placed in a 1-by-1 cm cube holder and placed at 5 cm from the smartphone, which acted as both the excitation source and signal-detecting device. For persistent decay measurement, first, the video recording was initiated, then the smartphone flashlight was put on for 5 s and then off while the video recording continued until all the persistent luminescent of the excited phosphor was completely exhausted. The measurement was repeated several times for each of the phosphors and as recorded videos processed in MATLAB to determine the persistent luminescence decay from the pixel region of interest of the phosphors. The green and red persistent lifetime of the blended phosphors was determined by splitting the channels from the raw video.

## Supplementary Information

Supplementary Information.
