# Supplementary material for: Dual-color dynamic anti-counterfeiting labels with persistent emission after visible excitation allowing smartphone authentication
Source: Sci Rep. 2022 Feb 8;12:2100. doi: 10.1038/s41598-022-05885-6 (PMC8826933; doi:10.1038/s41598-022-05885-6)
Supplement: Supplementary file 1 — Supplementary Information. [file 41598_2022_5885_MOESM1_ESM.docx]

**Dual-color dynamic anti-counterfeiting labels with persistent emission after visible excitation allowing smartphone authentication.**

Ngei Katumo, *^,1^ Kai Li, ^1^ Bryce S. Richards, ^1, 2^ Ian A. Howard ^*, 1, 2^

*^1^ Institute of Microstructure Technology, Karlsruhe Institute of Technology, Hermann-von-Helmholtz-Platz 1, 76344, Eggenstein-Leopoldshafen, Germany.*

*^2^ Light Technology Institute, Karlsruhe Institute of Technology, Engesserstrasse 13, 76131 Karlsruhe, Germany.*

**Supporting information**

Contents

[Experimental Section 3](#_Toc89333299)

[X-ray diffraction 3](#_Toc89333300)

[Scanning electron microscopy 3](#_Toc89333301)

[Photoexcitation spectra 3](#_Toc89333302)

[Results and discussion 4](#_Toc89333303)

[Photophysical properties of $\text{Sr}_{\text{1-x}}\text{S: }\text{Eu}_{\text{x}}^{\text{2+}}$ 4](#_Toc89333304)

[XRD patterns 5](#_Toc89333305)

[Scanning electron microscope images 8](#_Toc89333306)

[Determination of delayed lifetime fitting in the region-of-interest 9](#_Toc89333307)

[Photoluminescence (PL) excitation spectra of SAED phosphors 10](#_Toc89333308)

[Persistent lifetimes of SAED, $\text{Ca}_{\text{1-x}}\text{S: }\text{Eu}_{\text{x}}^{\text{2+}}$and $\text{Sr}_{\text{1-x}}\text{S: }\text{Eu}_{\text{x}}^{\text{2+}}$ phosphors 12](#_Toc89333309)

[Temperature-dependent persistent luminescence lifetime 15](#_Toc89333310)

[Temperature-dependent photoluminescence of $\text{Ca}_{\text{1-x}}\text{S: }\text{Eu}_{\text{x}}^{\text{2+}}\text{Sr}_{\text{1-x}}\text{S: }\text{Eu}_{\text{x}}^{\text{2+}}$ 18](#_Toc89333311)

[Optimizing $\text{Ca}_{\text{1-x}}\text{S: }\text{Eu}_{\text{x}}^{\text{2+}}\text{Sr}_{\text{1-x}}\text{S: }\text{Eu}_{\text{x}}^{\text{2+}}$color transition ratio 20](#_Toc89333312)

[Emoji labels temperature response 22](#_Toc89333313)

[References 23](#_Toc89333314)

# Experimental Section

X-ray diffraction
An X-ray powder diffractometer (XRD, Bruker D2 Phaser) with Cu K radiation (λ= 0.15405 Å) was used to investigate the phase and crystal structure of the phosphor materials in a wide range of Bragg angles (20^0^ ≤ 2Theta ≤ 70^0^).

## Scanning electron microscopy

The phosphor morphologies were studied using a scanning electron microscope (SEM, Zeiss Supra 90).

## Photoexcitation spectra

The excitation spectra were acquired using a spectrofluorometer (Varian Cary 50) in the 600-250 nm range. For these measurements, the phosphors were placed in 1 mm quartz cuvettes.

**Temperature-dependent photoluminescence and lifetime**

To have insights on the concentration quenching behavior of $\text{Ca}_{\text{1-x}}\text{S: }\text{Eu}_{\text{x}}^{\text{2+}}$ and $\text{Sr}_{\text{1-x}}\text{S:} \text{Eu}_{\text{x}}^{\text{2+}}$ phosphors, the samples were subjected to temperature-dependent photoluminescence and lifetime measurements. To acquire the data, a temperature-controlled thermal stage (MHCS622-V/G, Microptik) integrated with a high-resolution (0.1 K) temperature controller (MTDC600, Microptik) and a liquid nitrogen cooling system (LN2-SYS, Microptik) were used. The phosphors were filled in small alumina crucibles and placed on the heating stage chamber where the temperature could be varied from 100 K to 870 K. For the PL measurements, the emission of the phosphor following 450 nm excitation was collected with an optical fiber of 0.6-mm diameter (P/N78277, Newport) coupled with a spectrometer (CCS200, Thorlabs). To obtain the persistent luminescence decay curves, a smartphone (Samsung Galaxy A5 (2017)) was used to acquire the videos following excitation. The persistent luminescence decay curves were then derived from the analysis of the smartphone videos in Matlab where the average pixel intensity in the region of interest was computed as a function of time. The persistent luminescence lifetime was then fitted in the region of interest as shown in Figure S6.

# Results and discussion

## Photophysical properties of $\text{Sr}_{\text{1-x}}\text{S: }\text{Eu}_{\text{x}}^{\text{2+}}$

**(b)**

**(a)**

**Figure S1. Photoluminescence (PL) characteristics of** $\text{Sr}_{\text{1-x}}\text{S: }\text{Eu}_{\text{x}}^{\text{2+}}$ (a) The PLE (monitored at 614 nm) showing a broadband excitation region extending beyond 600 nm and the PL of $\text{Sr}_{\text{1-x}}\text{S: }\text{Eu}_{\text{x}}^{\text{2+}}$centered at 614 nm. Excitation of the broadband absorption with a smartphone flash led to the observed PL emission centered at 614 nm. A representative smartphone flashlight spectrum (black) is presented to demonstrate the excitation cross-section covered with the smartphone flash (b) The steady state photoluminescence quantum yield (PLQY) and delayed lifetime values of $\text{Sr}_{\text{1-x}}\text{S: }\text{Eu}_{\text{x}}^{\text{2+}}$ samples for 0.001 ≤ x ≤ 0.015 mol after 450 nm LED excitation. The PLQY reduces from ~39 % to ~28 %. Also provided are the persistent luminescence decay data that reduces from 377 ms to 150 ms after smartphone flashlight excitation for 5 s. The insets in (a) show the phosphor emissions after the smartphone excitation.

## XRD patterns

The XRD patterns of the SAED phosphors are as shown in Figure S2. The patterns revealed that the phosphors were polycrystalline. This is due to the solid-state-reaction synthesis procedure for these phosphors. In addition, no new peaks are observed because of the re-firing of the phosphors in the air. However, the peak intensities slightly decreased with the increase in re-firing temperature. This is usually due to crystal phase deterioration.^1^ The patterns were mapped to PDF. No. 00-034-0379. The zoomed-in region 20-30° revealed that upon refiring the phosphors in the air the FWHM increased. For the main peak, centered at 28.4° for the SAED(Ref) the FWHM was varied insignificantly from 0.10 to 0.13 for the SAED (700) phosphor. The increase is attributed to slight deterioration of the main crystal phase as the refiring in air temperature was increased. No secondary phases arose because of annealing the SAED phosphors in the air.

**Figure S2.** The XRD patterns for SAED phosphors. There are no special peaks arising from the re-firing of SAED in air.


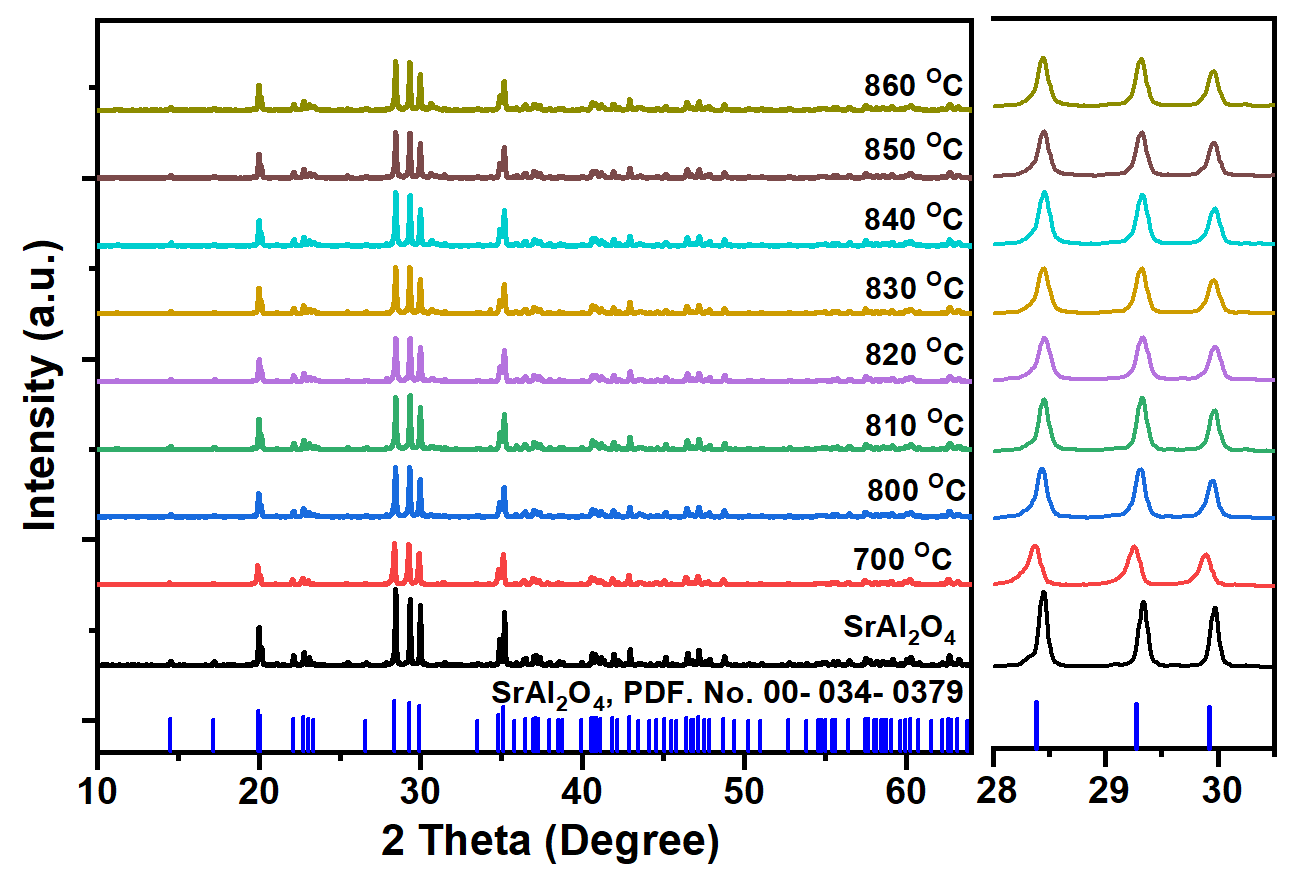


The XRD patterns of $\text{Ca}_{\text{1-x}}\text{S: }\text{Eu}_{\text{x}}^{\text{2+}}$ are shown in Figure S3. The CaS was mapped to a NaCl-type face-centered cubic structure in the Inorganic Crystal Structures, ICSD database, and coincided with ICSD card number 619538 and space group Fm-3m (225).^2^ For the $\text{Ca}_{\text{1-x}}\text{S:}\text{Eu}_{\text{x}}^{\text{2+}}$ the XRD patterns had sharp and well-defined diffraction peaks that indicated a reasonable degree of structural order and crystallinity. When the CaS host is doped with Eu^2+^ ions, it is expected that the Eu^2+^ ions ( ionic radius = 1.17 Å) replaces the Ca^2+^ (ionic radius 0.99 Å) lead to an expansion of the lattice.^3^ Figure S3 shows the systematic shift of the 222 peak to smaller angles in the XRD diffractogram as the Eu^2+^ concentration is increased. This is consistent with the expected lattice expansion in this case. Due to the relatively small volume fraction of the material affected by the replacement of Ca^2+^ with Eu^2+^ the shift in the lower angle peaks is not larger than the experimental uncertainty. Also the FWHM of the peaks remains unchanged to within the experimental uncertainty.

The XRD patterns of $\text{Sr}_{\text{1-x}}\text{S: }\text{Eu}_{\text{x}}^{\text{2+}}$ are shown in Figure S4. The patterns were mapped to the ICSD card number 28900 and had a face-centered cubic structure.^4^ The XRD patterns for all the samples had sharp and well-defined diffraction peaks and hence had a high degree of structural order and crystallinity. The FWHM observed at 2theta = 29.66^0^ peak was 0.14. Unlike in the CaS host case, no systematic shift in the peaks and FWHM are observed, as shown in the zoomed image in Figure S4. This is due to the comparable ionic radius of Eu^2+^ and Sr^2+^ which are 1.20 Å and 1.18 Å respectively.^5^


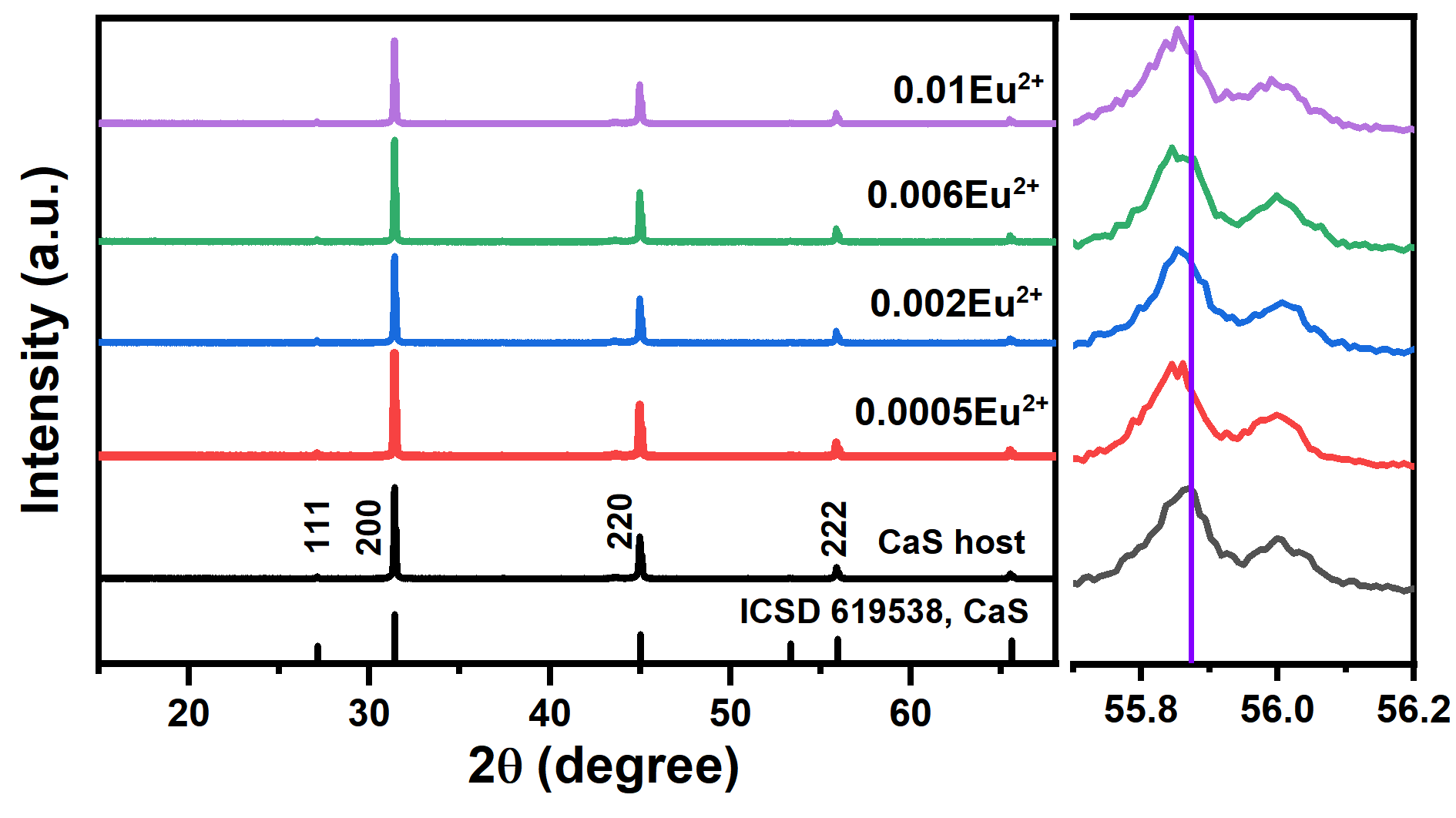


**Figure S3** The XRD patterns for $\text{Ca}_{\text{1-x}}\text{S: }\text{Eu}_{\text{x}}^{\text{2+}}$ phosphors. No special peaks arising from increased Eu^2+^ doping is observed. A slight shift to low 2theta degree is observed in the zoomed in 222 plane at 55.87^0^

**Figure S4.** The XRD patterns for $\text{Sr}_{\text{1-x}}\text{S: }\text{Eu}_{\text{x}}^{\text{2+}}$ phosphors. No special peaks arising from increased Eu^2+^ doping is observed.


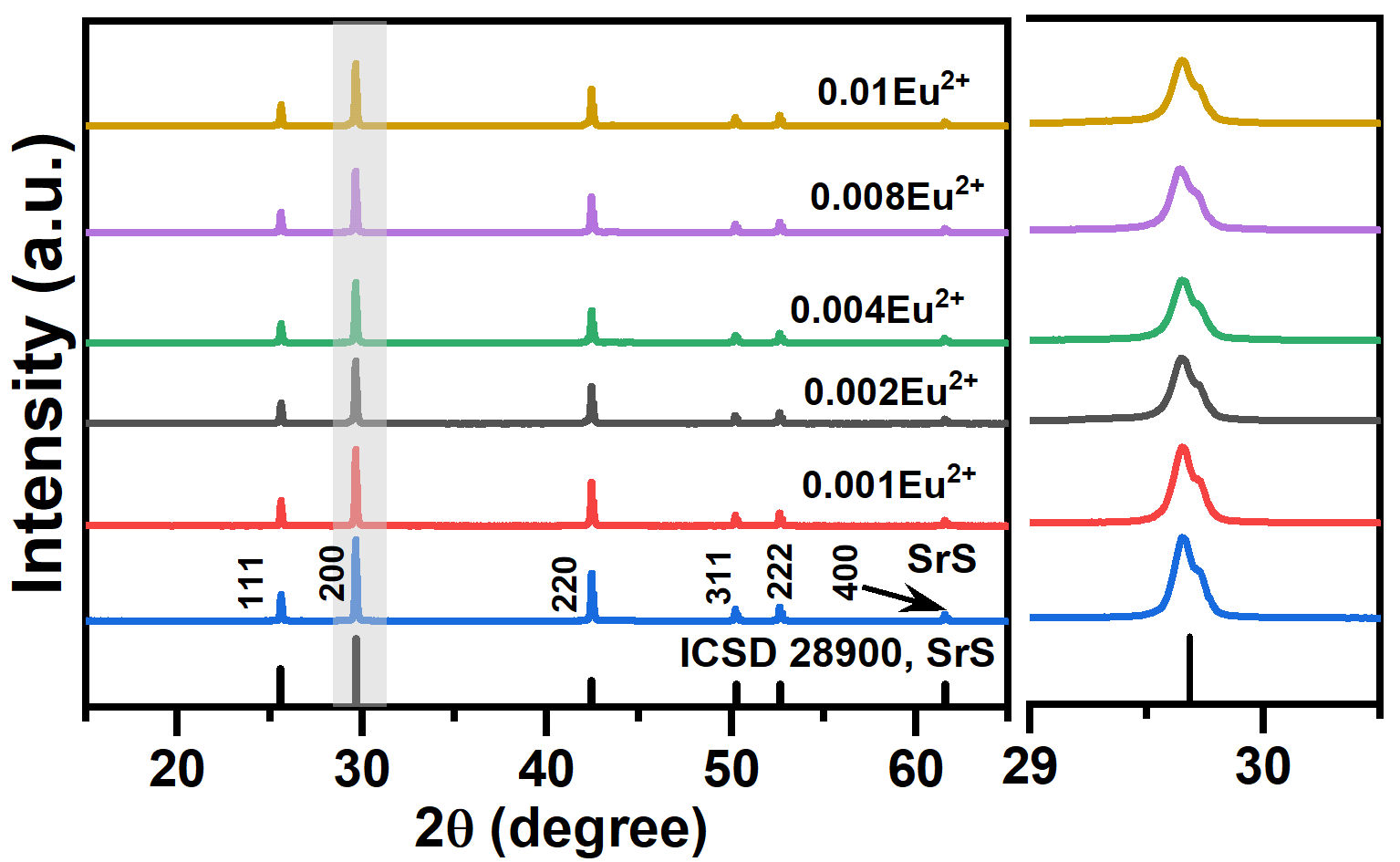


## Scanning electron microscope images

The SAED SEM images demonstrate that the phosphors were of irregular shapes and large grains; with most of the particles above 20 micrometers as shown in Figure S5 (a). They exhibited a high degree of agglomeration. The $\text{Ca}_{\text{1-x}}\text{S: }\text{Eu}_{\text{x}}^{\text{2+}}$and $\text{Sr}_{\text{1-x}}\text{S: }\text{Eu}_{\text{x}}^{\text{2+}}\text{ }$ exhibited quasi-spherical particles that agglomerated to larger grains as shown in Figure S5 (b) and Figure S5 (c). However, most of the individual grains were in the range of 1-10 µm.


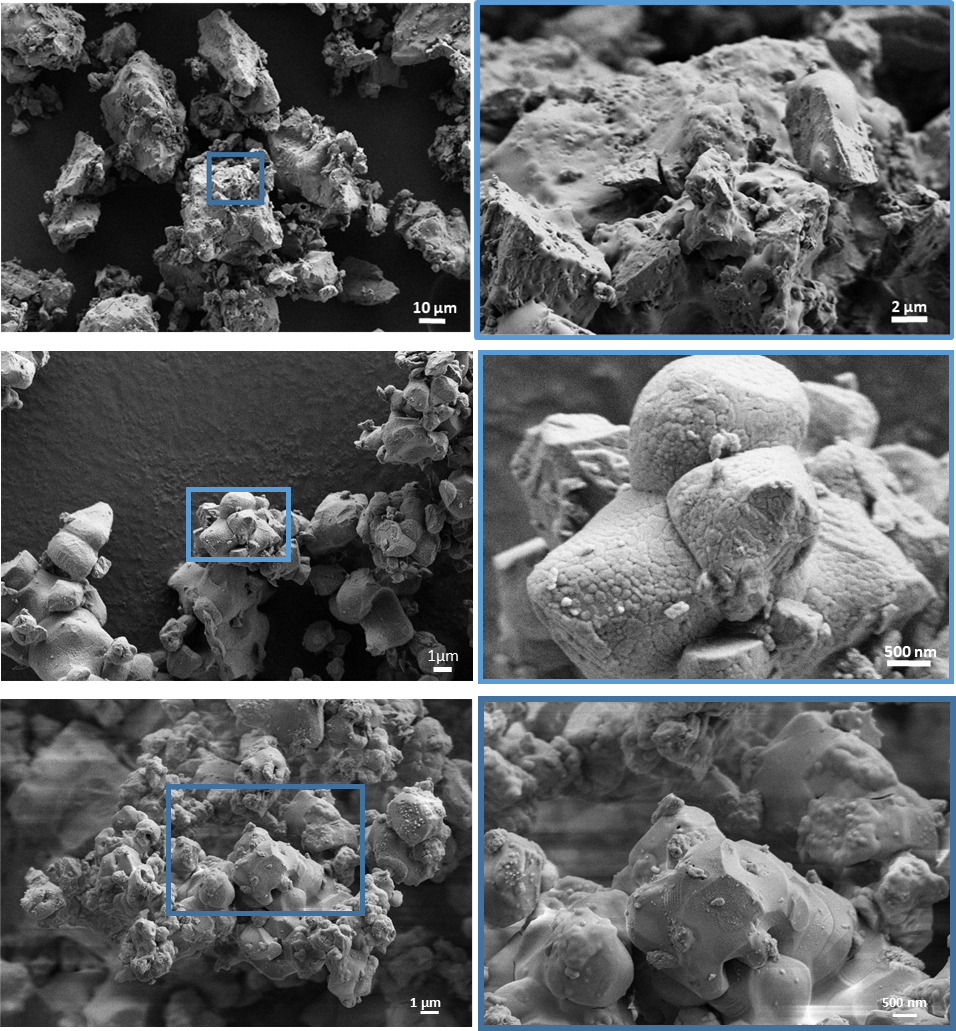


**(a)**

**(b)**

**(c)**

**Figure S5.** The SEM images of the phosphors (a) SAED (000). (b) $\text{Ca}_{\text{1-x}}\text{S: }\text{Eu}_{\text{x}}^{\text{2+}}$and (c) $\text{Sr}_{\text{1-x}}\text{S: }\text{Eu}_{\text{x}}^{\text{2+}}$ .The blue rectangles depict the magnified region in the right side that is also enclosed in a rectangle.

## Determination of delayed lifetime fitting in the region-of-interest

The SAED, $\text{Ca}_{\text{1-x}}\text{S: }\text{Eu}_{\text{x}}^{\text{2+}}$, and $\text{Sr}_{\text{1-x}}\text{S: }\text{Eu}_{\text{x}}^{\text{2+}}\text{ }$phosphors exhibited persistent luminescence and were excitable with a smartphone flashlight. We, therefore, identified a region of fitting, defined by boundaries t_1_ and t_2_ analogous to our previous work.^6^ The first bounder t_1_ was globally taken to be 0.2 s after the excitation source was put off, while the second boundary t_2_ was taken to be the time coinciding with the point at which the phosphor has exhausted 90% of its emission. The mark t_0_ is the time when the smartphone flashlight is switched off. This was computed by calculating the integral area of the emission as a function of time as shown in Figures S6. Since different phosphors have different persistent decay trends, the value t_2_ was therefore changing for each phosphor. By using the same procedure, we were able to determine the persistent luminescence decays of SAED, $\text{Ca}_{\text{1-x}}\text{S: }\text{Eu}_{\text{x}}^{\text{2+}}$, and $\text{Sr}_{\text{1-x}}\text{S: }\text{Eu}_{\text{x}}^{\text{2+}}\text{ }$as shown in Figure S9 - S12 and S15 for the blended phosphors respectively.


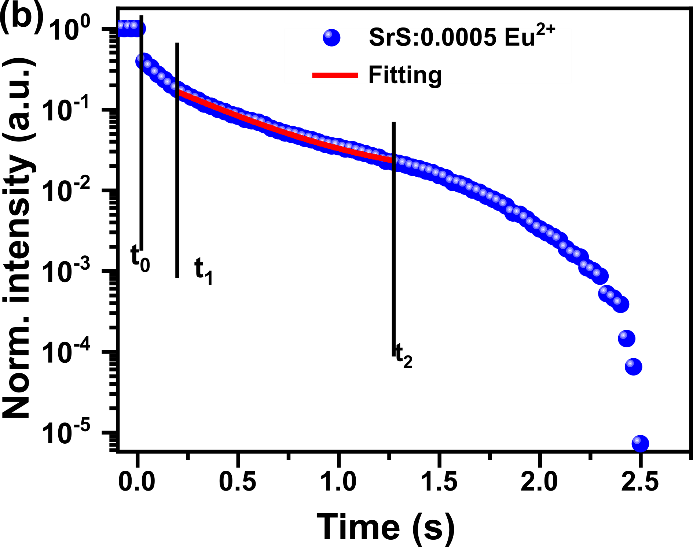

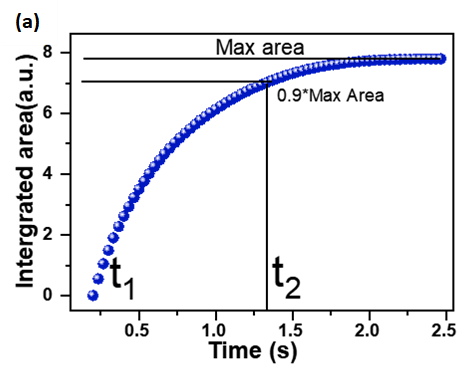


**Figure S6.** Determination of fitting region and persistent lifetime fitting. (a) Computed integral area of the delayed persistent emission of delayed emission of $\text{Sr}_{\text{0.985}}\text{S: }\text{Eu}_{\text{0.015}}^{\text{2+}}\text{ }$as a function of time to identify boundary time t_2_ at 90% emission exhaustion (b) Representative fitting in the t_1_ and t_2_ region under for bi-exponential and single exponential equations of the phosphors independently.


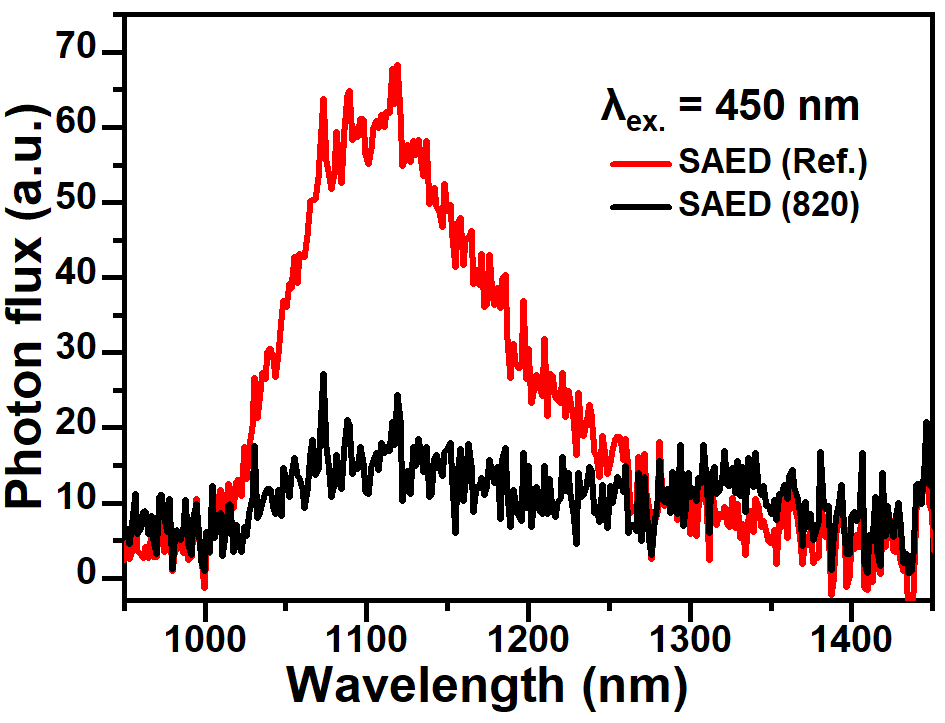


**Figure S7** Near infrared photoluminescence emission spectra of SAED (Ref.) and SAED annealed at 820°C after 450 nm excitation.

## Photoluminescence (PL) excitation spectra of SAED phosphors

The SAED phosphors annealed in air for an hour at various temperature ranges experienced narrowing of the absorption band as monitored from the emission peak at 525 nm as shown in Figure S7. The narrowing of the absorption lead that is due to the 4f^7^ –  4f^6^5d^1^ transition of the Eu^2+^ is attributed to the oxidation of Eu^2+^ to a non-radiative Eu^3+^ ion.^1,7^ Annealing the SAED phosphors in air leads to the formation of a non-radiative Eu^3+^ that effectively reduces the trap density and depth. As a result, persistent luminescence and the emission intensity after excitation decreases. It is also worth noting that there is reduced excitation area using a smartphone because of the narrowing of the SAED absorption band.

**Figure S8** The PLE of SAED 500-250 nm showing the narrowing of the absorption band as the annealing in air. The absorption band narrows down with the increase in annealing in air temperature.

## **Persistent lifetimes of SAED,** $\text{Ca}_{\text{1-x}}\text{S: }\text{Eu}_{\text{x}}^{\text{2+}}$ **and** $\text{Sr}_{\text{1-x}}\text{S: }\text{Eu}_{\text{x}}^{\text{2+}}$ **phosphors**

**Figure S9** Persistent luminescence decay lifetime of $\text{Sr}_{\text{1-x}}\text{S: }\text{Eu}_{\text{x}}^{\text{2+}}$ phosphors extracted from the analysis of videos acquired using a smartphone following 5 s excitation with the smartphone-flashlight. The data demonstrates that the persistent luminescence lifetime decreases with increase in Eu^2+^ doping

**Figure S10** Persistent decay of SAED phosphors annealed at 000 - 860 ^0^C for one hour in air. Re-firing the phosphors in air decreased the persistent luminescence decay of the SAED phosphors. For phosphors annealed at 820^0^C and below, a double exponential fitting was used while phosphors annealed in air at 820^0^C and above the persistent decay were fitted using a single exponential.

**Figure S11** Persistent luminescence decay lifetime of $\text{Ca}_{\text{1-x}}\text{S: }\text{Eu}_{\text{x}}^{\text{2+}}$ phosphors extracted from the analysis of videos acquired using a smartphone following 5 s excitation with the smartphone-flashlight. The data demonstrates that the persistent luminescence lifetime decreases with increase in Eu^2+^ doping.

**Table S1.** Persistent luminescence decay lifetime fitting parameters of SAED phosphors after 5 s smartphone flashlight excitation. The persistent luminescence of the SAED phosphors decreased with an increase in the refiring temperature in air.

| Phosphor | Temp. (^0^C) | Y_0_ | A_1_ | $\boldsymbol{\tau}_{\boldsymbol{1}}$(s) | A_2_ | $\boldsymbol{\tau}_{\boldsymbol{2}}\boldsymbol{(s)}$ | $\boldsymbol{\tau}_{\boldsymbol{avg.}}$ (s) |
| --- | --- | --- | --- | --- | --- | --- | --- |
| SAED (000) | 0 | 0.04 | 0.23 | 2.07 | 0.32 | 12.89 | 11.74 |
| SAED (700) | 700 | 0.04 | 0.21 | 1.75 | 0.31 | 10.88 | 9.97 |
| SAED (800) | 800 | 0.00 | 0.27 | 0.71 | 0.26 | 8.47 | 7.85 |
| SAED (810) | 810 | 0.00 | 0.22 | 1.01 | 0.23 | 4.88 | 4.25 |
| SAED (820) | 820 | 0.00 | 0.18 | 0.69 | 0.23 | 2.95 | 2.61 |
| SAED (830) | 830 | 0.00 | 0.08 | 0.26 | 0.14 | 1.74 | 1.62 |
| SAED (840) | 840 | 0.00 | 0.00 | 0.00 | 0.18 | 0.88 | 0.88 |
| SAED (850) | 850 | 0.00 | 0.00 | 0.00 | 0.13 | 0.62 | 0.62 |
| SAED (860) | 860 | 0.00 | 0.00 | 0.00 | 0.12 | 0.50 | 0.50 |

## Temperature-dependent persistent luminescence lifetime

The temperature-dependent persistent lifetime for$\text{ }\text{Ca}_{\text{0.999}}\text{S: }\text{Eu}_{\text{0.001}}^{\text{2+}}$, $\text{Ca}_{\text{0.996}}\text{S: }\text{Eu}_{\text{0.004}}^{\text{2+}}\text{ }$and $\text{Ca}_{\text{0.992}}\text{S: }\text{Eu}_{\text{0.008}}^{\text{2+}}$ are shown in Figure S12(a-c). For all the samples, the maximum persistent luminescence was registered at around 233K, but a slight persistent decay is observed at 213K. Further increase in temperature beyond 233K leads to a gradual decrease in persistent decay. The persistent luminescence decay is fitted using a single exponential equation of the form:

$I=I_{o}+A*exp({-t}/{\tau)}$ (S1)


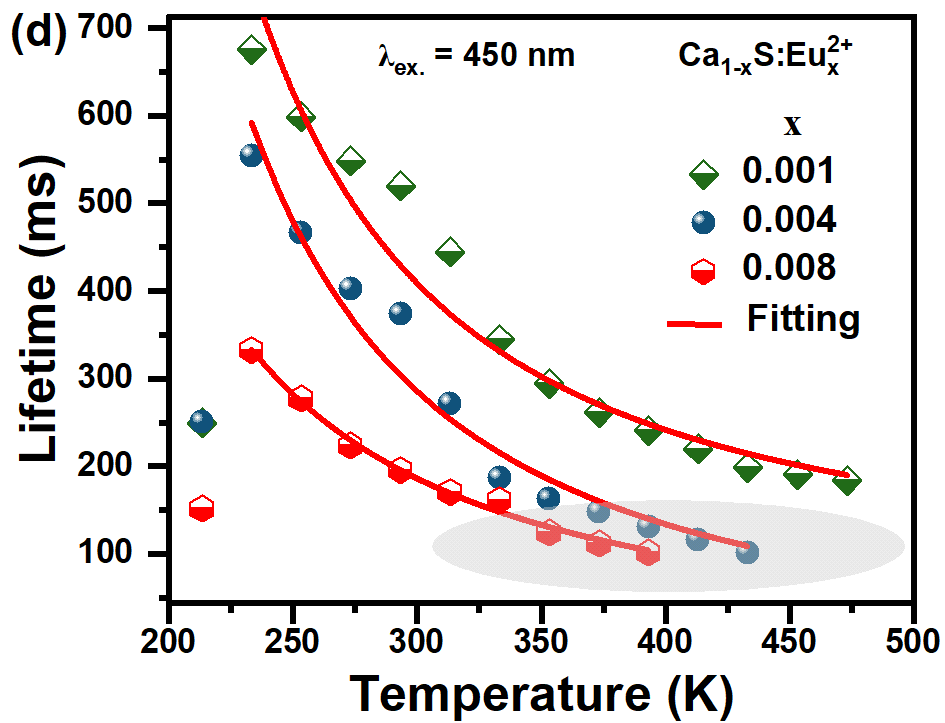

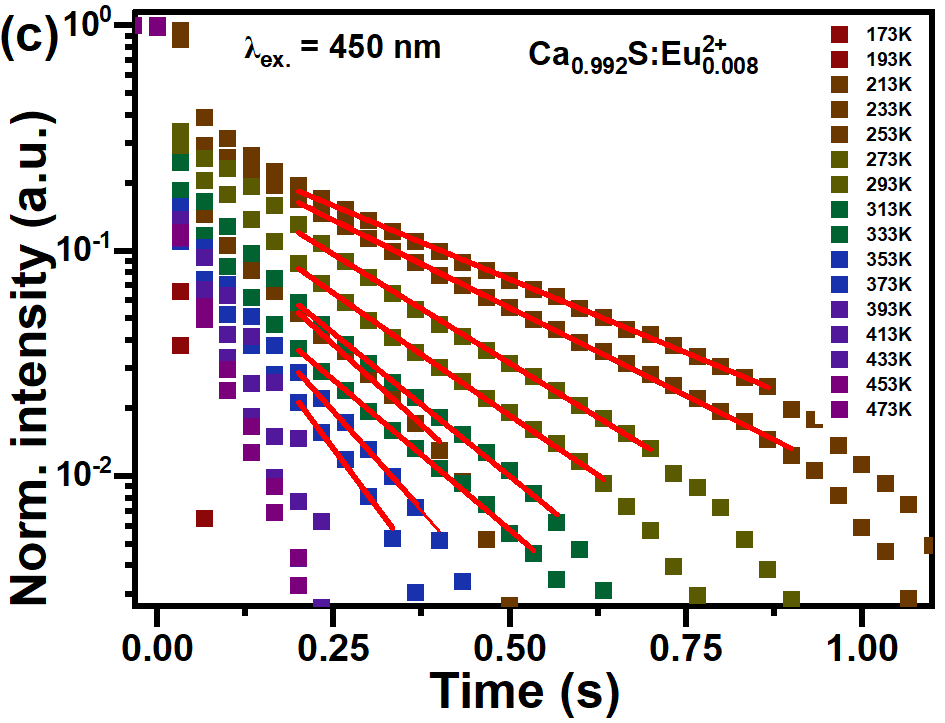

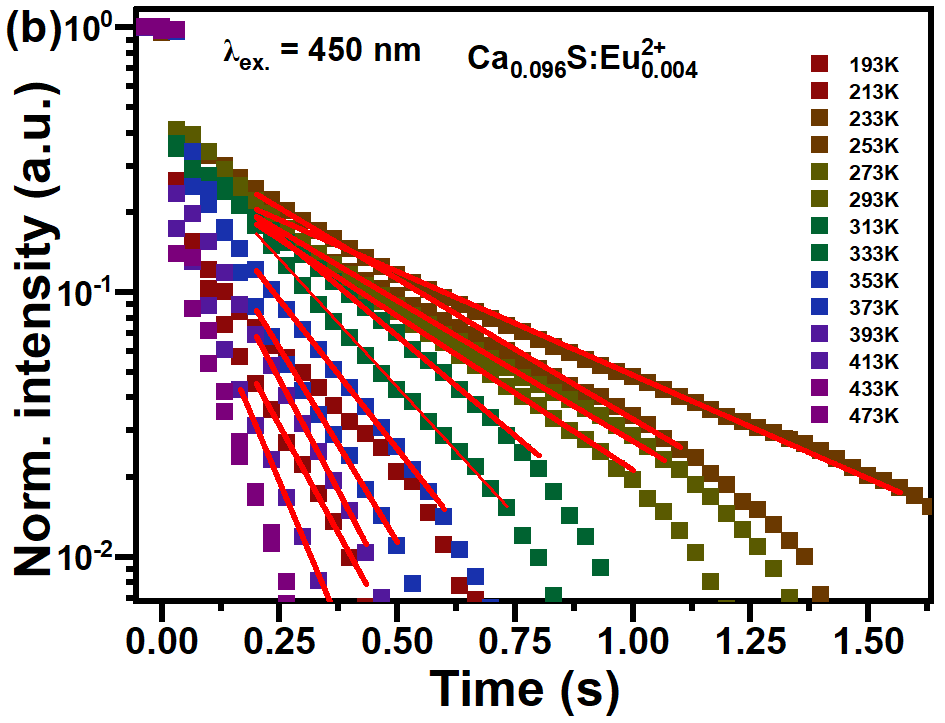

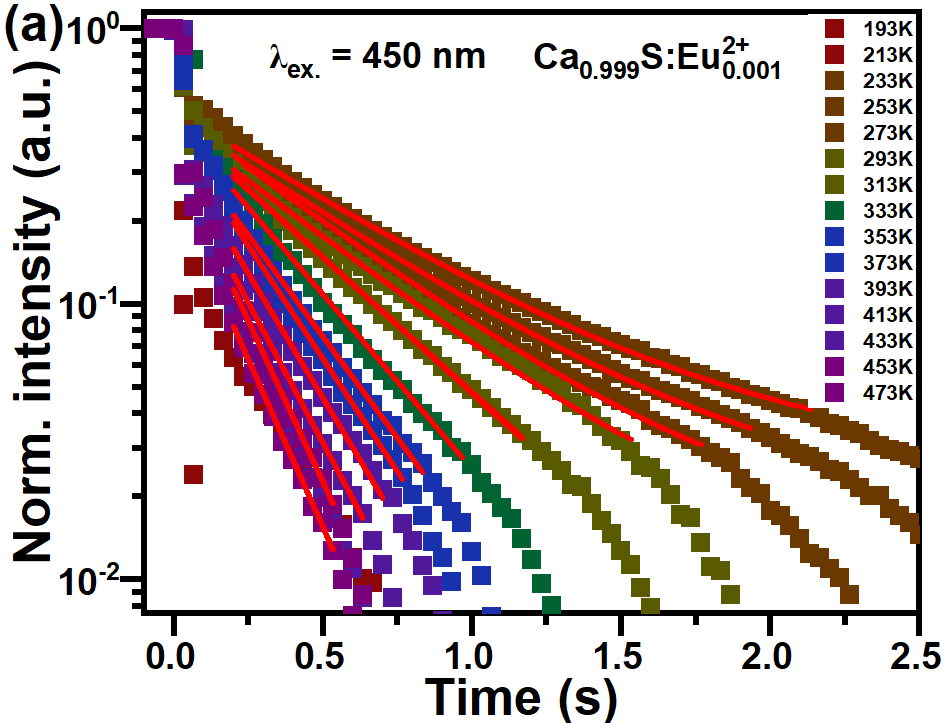


**Figure S12.** The temperature dependent persistent luminescence lifetime of $\text{Ca}_{\text{1-x}}\text{S: }\text{Eu}_{\text{x}}^{\text{2+}}\text{ }$ phosphors (x = 0.001, 0.004 and 0.08) at various temperatures following 450 nm excitation. (a) The persistent luminescence lifetime of $\text{Ca}_{\text{0.999}}\text{S: }\text{Eu}_{\text{0.001}}^{\text{2+}}$ in the 193- 473 K. (b) The persistent luminescence lifetime $\text{Ca}_{\text{0.996}}\text{S: }\text{Eu}_{\text{0.004}}^{\text{2+}}\text{ }$ in the 193- 473 K and (c) the persistent luminescence lifetime of $\text{Ca}_{\text{0.992}}\text{S: }\text{Eu}_{\text{0.008}}^{\text{2+}}\text{ }$phosphor for the 173 - 473 K. (d) Summary of the integral emission of the phosphors in the 193 -473 K range.

where 𝜏 is the lifetime, A is a constant coefficient and I_0_ is an offset. A summary of the obtained lifetime values of the three samples is shown in Figure S12(d). The persistent lifetime as a function of temperature can be estimated within a simple Arrhenius model as:

$\tau(T)=A*exp({-E_{a}}/{k_{B}T)}$ (S2)

Where $\tau$ is the measured lifetime, A is a constant, $k_{B}$ is Boltzman constant and $E_{a}$ is the activation energy corresponding to the trap depth. From the persistent luminescence data, the apparent activation energy is estimated to be 0.07 eV and slightly reduced to 0.05 eV for $\text{Ca}_{\text{0.999}}\text{S: }\text{Eu}_{\text{0.001}}^{\text{2+}}$ and $\text{Ca}_{\text{0.992}}\text{S: }\text{Eu}_{\text{0.008}}^{\text{2+}}$ respectively. The trap depth stays the same across the Eu^2+^ concentrations. This ruling out that a change in trap depth is responsible for the persistent lifetime tuning with Ca^2+^ concentration suggests that the change in persistent lifetime is due to a change in the nonradiative deactivation of the trapped state as a function of the Eu^2+^ concentration.

## Temperature-dependent photoluminescence of $\text{Ca}_{\text{1-x}}\text{S: }\text{Eu}_{\text{x}}^{\text{2+}}$, and $\text{Sr}_{\text{1-x}}\text{S: }\text{Eu}_{\text{x}}^{\text{2+}}$,

To investigate why the persistent luminescence lifetime and PLQY decrease with an increase in Eu^2+^ concentration, temperature-dependent photoluminescence studies were performed. Figure S13 shows the temperature-dependent photoluminescence (TDPL) of$\text{ Ca}_{\text{0.999}}\text{S: }\text{Eu}_{\text{0.001}}^{\text{2+}}$, $\text{Ca}_{\text{0.996}}\text{S: }\text{Eu}_{\text{0.004}}^{\text{2+}}\text{ }$and $\text{Ca}_{\text{0.992}}\text{S: }\text{Eu}_{\text{0.008}}^{\text{2+}}$ in the 100 - 823K region. Figures S13 (a-c) show that the integral emission decreased with an increase in temperature from 113 K to 823 K. Overall the emission decreases as the temperature is increased for all three samples. This is also accompanied by a blue shift of the emission as the temperature is increased. The integral emission is computed as a function of temperature. From the integral emission summary in Figure S12(d) it’s clear that the emission quenching with respect to temperature increases with an increase in Eu^2+^ doping. The more significant quenching suggests more easily activated non-radiative rates for the higher Eu^2+^ concentrations, that could act to also deactivate the traps responsible for the persistent emission and possibly explain the reduction of persistent lifetime with increasing Eu^2+^ concentration.


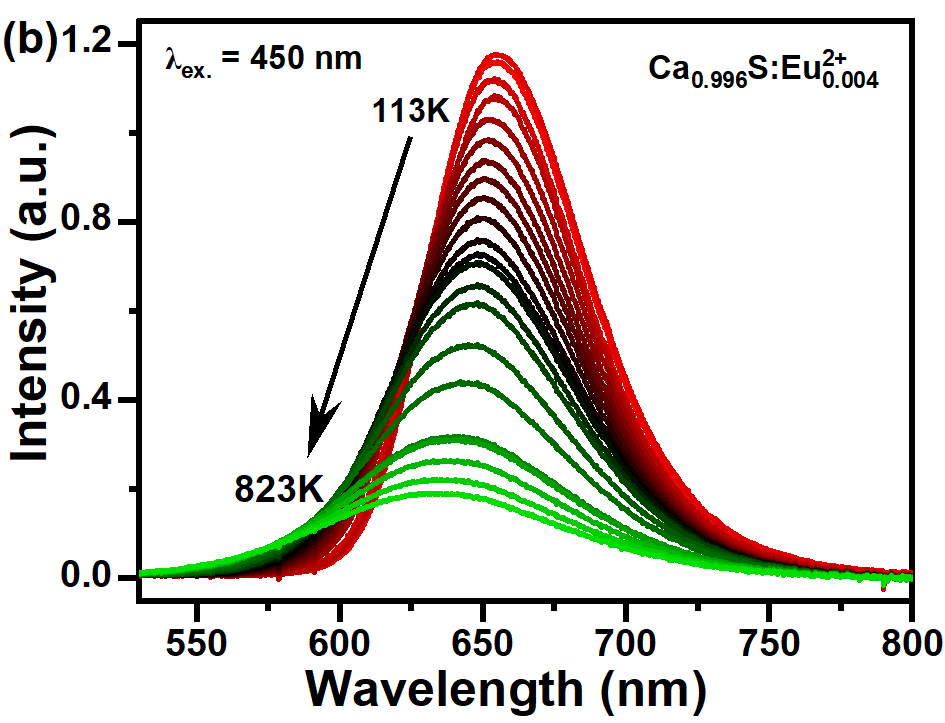

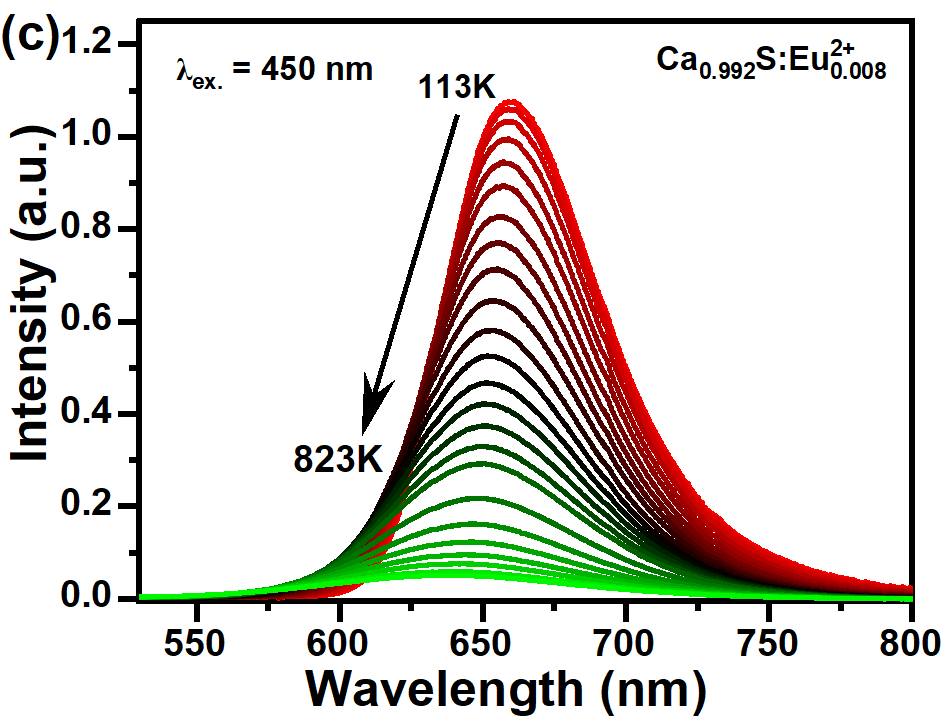

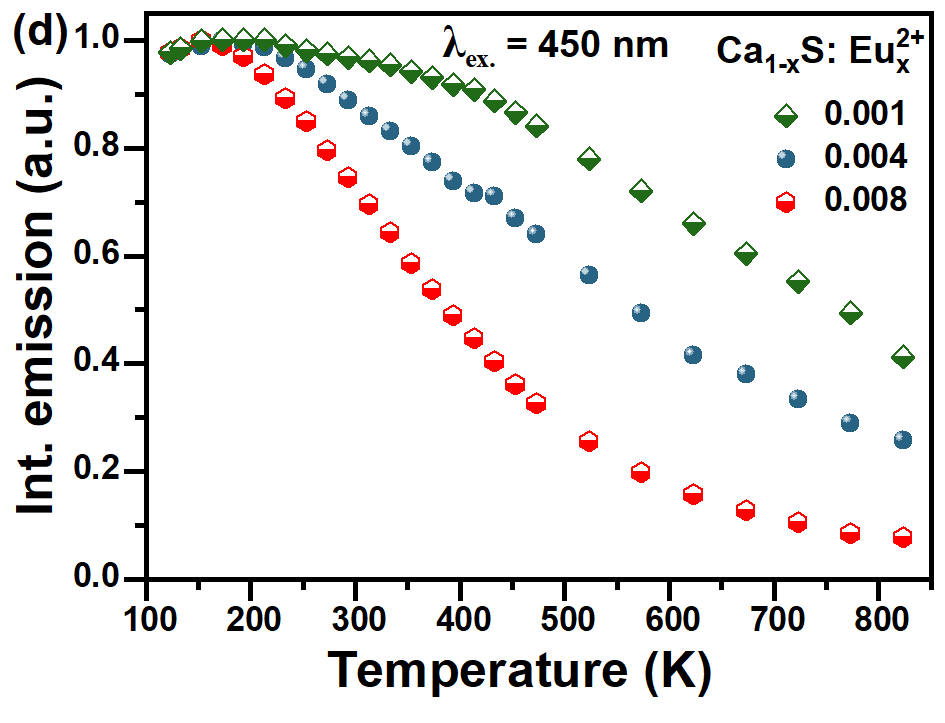

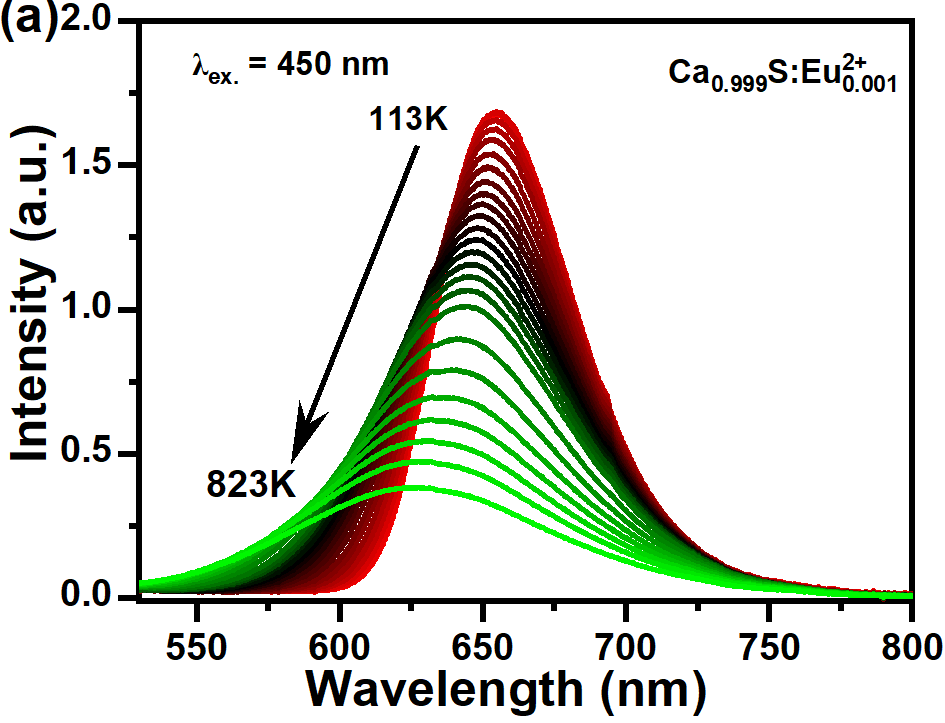


**Figure S13** Temperature dependent photoluminescence emission in the 100-823 K range under 450 nm excitation. (a) The$\text{ }\text{Ca}_{\text{0.999}}\text{S: }\text{Eu}_{\text{0.001}}^{\text{2+}}\text{phosphor}$. (b) The $\text{Ca}_{\text{0.996}}\text{S: }\text{Eu}_{\text{0.004}}^{\text{2+}}\text{ }$ phosphor. (c) The $\text{Ca}_{\text{0.992}}\text{S: }\text{Eu}_{\text{0.008}}^{\text{2+}}\text{ }$ phosphor. (d) Summary of the integral emission of the phosphors in the 100-823 K range.

## Optimizing $\text{Ca}_{\text{1-x}}\text{S: }\text{Eu}_{\text{x}}^{\text{2+}}$, and $\text{Sr}_{\text{1-x}}\text{S: }\text{Eu}_{\text{x}}^{\text{2+}}$ color transition ratio


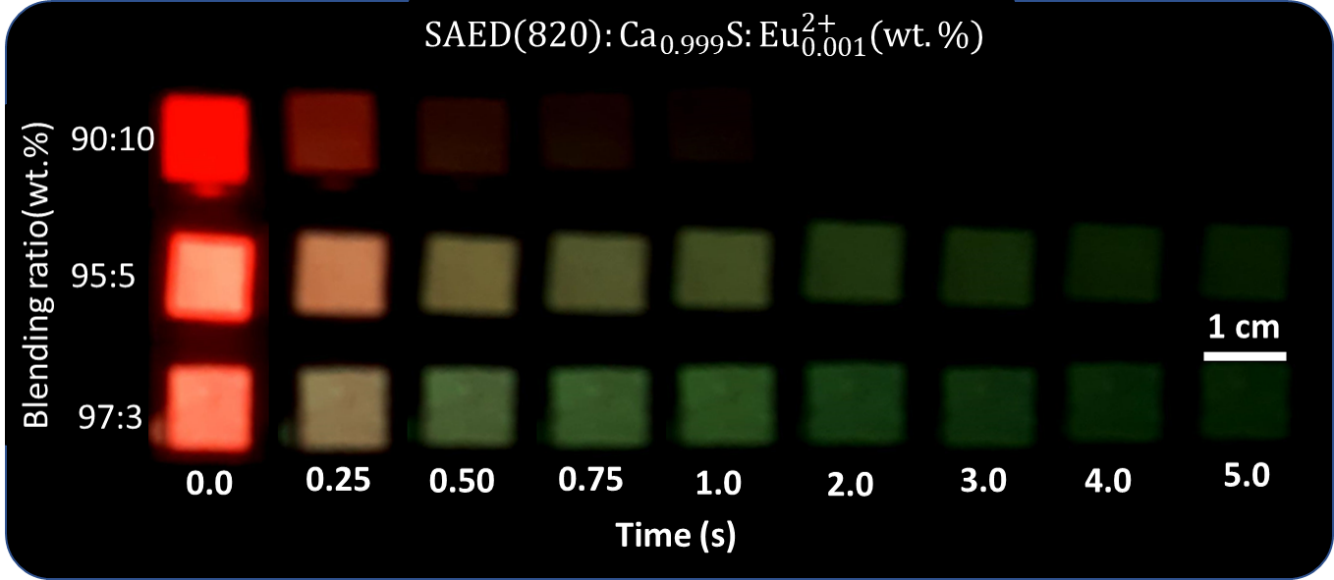


**Figure S14.** Perceived color change from red to green depending on the blending ratios using SAED(820): $\text{Ca}_{\text{0.999}}\text{S:}\text{Eu}_{\text{0.001}}^{\text{2+}}$. For 90:10 blending ratio ratio little excitation reaches the SAED that green emission is not observed. For 95:5, the transition is gradual as both the CaS and SAED are well excited by the illumination. For the 97:3, the green emission from the SAED overwhelms the CaS emission rather soon, leading the delayed red to be hardly percieved. Given the apparent balance and most radual percieved transition 95:5 is presented in the main text. The percieved color transition depends on the fraction of excitation photons absorbed in each material, which could also change with powder thickness if the powder is not sufficiently thick to be optically dense (no transmision). For this work all powder layers were 1 mm thick and optically dense such that minor variations in thickness did not affect the ratio of the red to green emission.

**Figure S15.** The extracted red and green channels persistent lifetime of SAED (830): $\text{Ca}_{\text{0.994}}\text{S: }\text{Eu}_{\text{0.006}}^{\text{2+}}$ and SAED (830): $\text{Sr}_{\text{0.985}}\text{S: }\text{Eu}_{\text{0.015}}^{\text{2+}}$ phosphors blend after smartphone flashlight excitation. The persistent lifetime decays are plotted alongside the lifetimes of the pure phosphors.


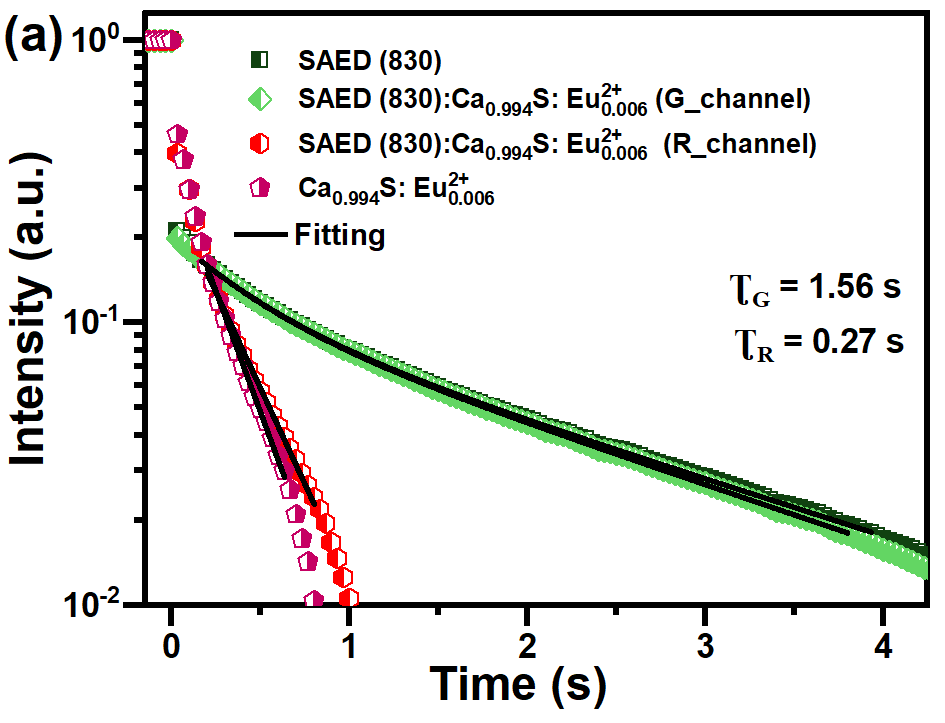

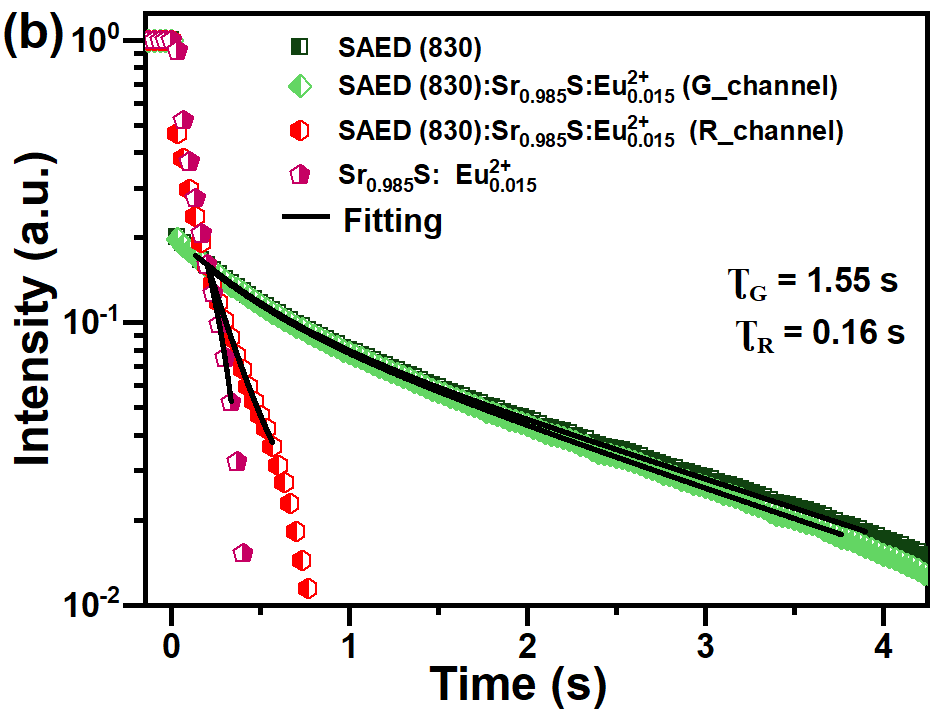


**Table S 2.** Persistent luminescence lifetime fitting parameters of the red and green channels of phosphor blends following 5 s of smartphone flashlight excitation.

| Phosphor blend (95:5 wt./wt.) | Channel | Y_0_ | A_1_ | $\boldsymbol{\tau}_{\boldsymbol{1}}$(s) | A_2_ | $\boldsymbol{\tau}_{\boldsymbol{2}}\boldsymbol{(s)}$ | $\boldsymbol{\tau}_{\boldsymbol{avg.}}$ (s) |
| --- | --- | --- | --- | --- | --- | --- | --- |
| SAED (820): $\text{Ca}_{\text{0.9995}}\text{S: }\text{Eu}_{\text{0.0005}}^{\text{2+}}$ | Green | 0.0 | 0.15 | 0.54 | 0.21 | 2.83 | 2.56 |
|  | Red | 0.0 | 0.0 | 0.0 | 0.50 | 0.60 | 0.60 |
| SAED (820): $\text{Ca}_{\text{0.996}}\text{S: }\text{Eu}_{\text{0.004}}^{\text{2+}}$ | Green | 0.0 | 0.14 | 0.54 | 0.23 | 2.81 | 2.54 |
|  | Red | 0.0 | 0.0 | 0.0 | 0.44 | 0.40 | 0.40 |
| SAED (820): $\text{Sr}_{\text{0.992}}\text{S: }\text{Eu}_{\text{0.008}}^{\text{2+}}$ | Green | 0.0 | 0.15 | 0.36 | 0.20 | 2.77 | 2.55 |
|  | Red | 0.0 | 0.0 | 0.0 | 0.70 | 0.23 | 0.23 |
| SAED (820): $\text{Sr}_{\text{0.985}}\text{S: }\text{Eu}_{\text{0.015}}^{\text{2+}}$ | Green | 0.0 | 0.15 | 0.34 | 0.22 | 2.73 | 2.56 |
|  | Red | 0.0 | 0.0 | 0.0 | 0.0 | 0.78 | 0.154 |
| SAED (830): $\text{Ca}_{\text{0.994}}\text{S: }\text{Eu}_{\text{0.006}}^{\text{2+}}$ | Green | 0.0 | 0.09 | 0.36 | 0.15 | 1.73 | 1.56 |
|  | Red | 0.0 | 0.0 | 0.0 | 0.28 | 0.27 | 0.27 |
| SAED (830): $\text{Sr}_{\text{0.985}}\text{S: }\text{Eu}_{\text{0.015}}^{\text{2+}}$ | Green | 0.0 | 0.08 | 0.31 | 0.12 | 1.70 | 1.55 |
|  | Red | 0.0 | 0.0 | 0.0 | 0.39 | 0.16 | 0.16 |

## Emoji labels temperature response


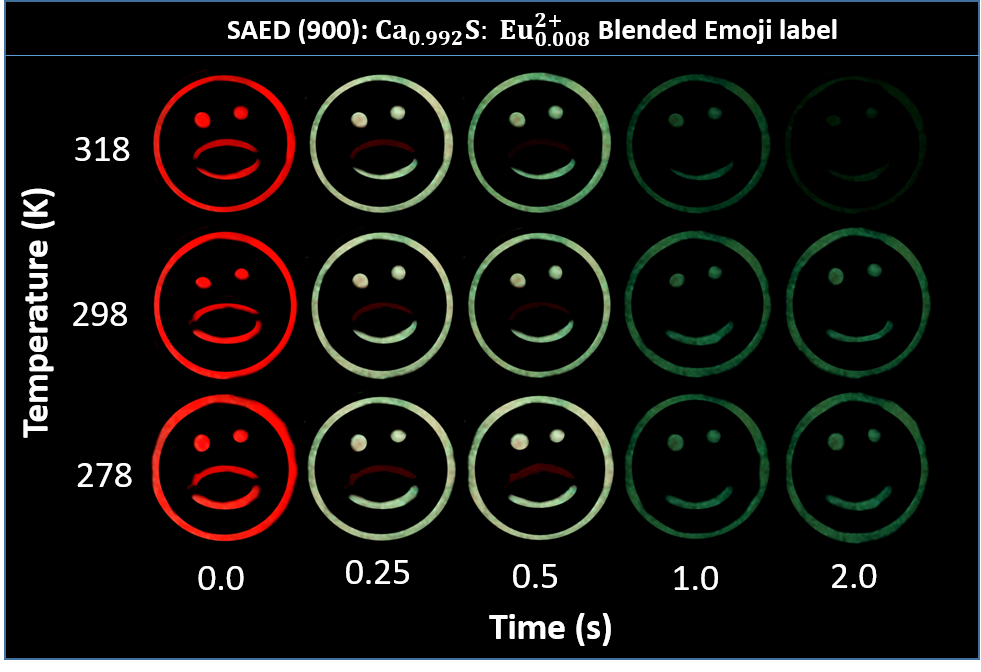


**Figure S 16** Persistent luminescent emoji images utilizing both color tunability and persistent emission to transform emoji from an initial wow image to smiley emoji after smartphone excitation for 10 s at three temperature settings. As the temperature is increased the transitions occurs faster but the same transition is observable at all temperatures.

**Figure S16** Persistent luminescent emoji images utilizing both color tunability and persistent emission to transform emoji from an initial wow image to smiley emoji after smartphone excitation for 10 s at three temperature settings. As the temperature is increased the transitions occurs faster but the same transition is observable at all temperatures.

The emoji label transition from red to green was investigated under three temperature settings as shown in Figure S16. At low temperature, 278 K, the Emoji remains bright for a longer time, and hence transitions are a bit longer compared to the room temperature transitions. On the other hand, at a higher temperature, the transition is fast, with the emission at 2.0 s almost being exhausted. These results show that the anti-counterfeiting label can sufficiently be used at various temperatures for color change.

# References

1 Karacaoglu, E. & Karasu, B. The effects of re-firing process under oxidizing atmosphere and temperatures on the properties of strontium aluminate phosphors. *Materials Research Bulletin* **48**, 3702-3706 (2013).

2 Petzel, T. Über die Darstellung von CaS, SrS und BaS aus den Metallen und Schwefelwasserstoff in flüssigem Ammoniak. *Zeitschrift für anorganische und allgemeine Chemie* **396**, 173-177 (1973).

3 Hu, Y. *et al.* Preparation and luminescent properties of (Ca_1-x_, Sr_x_) S: Eu^2+^ red-emitting phosphor for white LED. *J. Lumin.* **111**, 139-145 (2005).

4 Primak, W., Kaufman, H. & Ward, R. X-ray diffraction studies of systems involved in the preparation of alkaline earth sulfide and selenide phosphors1. *Journal of the American Chemical Society* **70**, 2043-2046 (1948).

5 Kim, D. Recent Developments in Lanthanide-Doped Alkaline Earth Aluminate Phosphors with Enhanced and Long-Persistent Luminescence. *Nanomaterials* **11**, 723 (2021).

6 Katumo, N. *et al.* Anticounterfeiting Labels with Smartphone‐Readable Dynamic Luminescent Patterns Based on Tailored Persistent Lifetimes in Gd_2_O_2_S: Eu^3+^/Ti^4+^. *Adv. Mater. Technol.* **6**, 2100047 (2021).

7 Havasi, V. *et al.* On the effects of milling and thermal regeneration on the luminescence properties of Eu2+ and Dy3+ doped strontium aluminate phosphors. *J. Lumin.* **219**, 116917 (2020).
